# Omics for prediction of environmental health effects: Blood leukocyte-based cross-omic profiling reliably predicts diseases associated with tobacco smoking

**DOI:** 10.1038/srep20544

**Published:** 2016-02-03

**Authors:** Panagiotis Georgiadis, Dennie G. Hebels, Ioannis Valavanis, Irene Liampa, Ingvar A. Bergdahl, Anders Johansson, Domenico Palli, Marc Chadeau-Hyam, Aristotelis Chatziioannou, Danyel G. J. Jennen, Julian Krauskopf, Marlon J. Jetten, Jos C. S. Kleinjans, Paolo Vineis, Soterios A. Kyrtopoulos, The EnviroGenomarkers consortium, Ralph Gottschalk, Danitsja van Leeuwen, Leen Timmermans, Theo M.C.M. de Kok, Maria Botsivali, Benedetta Bendinelli, Rachel Kelly, Roel Vermeulen, Lutzen Portengen, Fatemeh Saberi-Hosnijeh, Beatrice Melin, Göran Hallmans, Per Lenner, Hector C. Keun, Alexandros Siskos, Toby J. Athersuch, Manolis Kogevinas, Euripides G. Stephanou, Antonis Myridakis, Lucia Fazzo, Marco De Santis, Pietro Comba, Hannu Kiviranta, Panu Rantakokko, Riikka Airaksinen, Päivi Ruokojärvi, Mark Gilthorpe, Sarah Fleming, Thomas Fleming, Yu-Kang Tu, Bo Jonsson, Thomas Lundh, Wei J. Chen, Wen-Chung Lee, Chuhsing Kate Hsiao, Kuo-Liong Chien, Po-Hsiu Kuo, Hung Hung, Shu-Fen Liao

**Affiliations:** 1 National Hellenic Research Foundation, Institute of Biology, Medicinal Chemistry and Biotechnology, 48 Vas. Constantinou Ave., Athens 11635, Greece; 2 Maastricht University, Minderbroedersberg 4-6, 6211 LK, Maastricht, Netherlands; 3 Department of Biobank Research, and Occupational and Environmental Medicine, Department of Public Health and Clinical Medicine, Umeå University, Sweden; 4 Nutrition Research, Department of Public Health and Clinical Medicine, Umeå University, Sweden; 5 The Institute for Cancer Research and Prevention, Italy; 6 MRC-PHE Centre for Environment and Health, Department of Epidemiology and Biostatistics, School of Public Health, Faculty of Medicine, Imperial College London, St Mary’s Campus, Norfolk Place, W2 1PG, UK; 7 Division of Environmental Epidemiology, Institute for Risk Assessment Sciences, Utrecht University, The Netherlands; 8 Oncology, Department of Radiation Sciences, Umeå University, Sweden; 9 Biomolecular Medicine, Department of Surgery and Cancer, Faculty of Medicine, Imperial College London, Sir Alexander Fleming Building, South Kensington, London, SW7 2AZ, UK; 10 Centre for Research in Environmental Epidemiology (CREAL), Doctor Aiguader 88, 08003 Barcelona, Spain; 11 University of Crete, Heraklion, Greece; 12 Istituto Superiore di Sanita, Rome Italy; 13 National Institute for Health and Welfare, Kuopio, Finland; 14 University of Leeds, UK; 15 Lund University, Sweden; 16 National Taiwan University, Taipei, Taiwan

## Abstract

The utility of blood-based omic profiles for linking environmental exposures to their potential health effects was evaluated in 649 individuals, drawn from the general population, in relation to tobacco smoking, an exposure with well-characterised health effects. Using disease connectivity analysis, we found that the combination of smoking-modified, genome-wide gene (including miRNA) expression and DNA methylation profiles predicts with remarkable reliability most diseases and conditions independently known to be causally associated with smoking (indicative estimates of sensitivity and positive predictive value 94% and 84%, respectively). Bioinformatics analysis reveals the importance of a small number of smoking-modified, master-regulatory genes and suggest a central role for altered ubiquitination. The smoking-induced gene expression profiles overlap significantly with profiles present in blood cells of patients with lung cancer or coronary heart disease, diseases strongly associated with tobacco smoking. These results provide proof-of-principle support to the suggestion that omic profiling in peripheral blood has the potential of identifying early, disease-related perturbations caused by toxic exposures and may be a useful tool in hazard and risk assessment.

The relative insensitivity of epidemiological investigations for the detection of environmental and other health hazards and the quantification of associated risks underlines the need for novel *in vitro* and *in vivo* tools that enable the identification of early biological signals which can be used to predict future disease. Ongoing efforts in this direction focus on the characterization through *in vitro* testing, including toxicogenomic profiling, of biological pathways whose perturbation by chemicals leads to the manifestation of toxicity^1^, in combination with the search for relationships between gene expression profiles induced by chemicals with profiles associated with human diseases (disease connectivity mapping)^2^. A complementary approach towards the same goal which could be exploited in the context of population-based studies, including population biomonitoring, involves the identification of perturbations induced by environmental exposures in readily accessible human tissues and the characterisation of their relationship with disease pathogenesis. In this context a number of studies have examined the impact of various environmental exposures on different types of blood-based omic profiles in human populations, and their results in many cases support the notion that such profile changes reflect to some degree perturbations related to known or suspected toxic hazards associated with the exposures concerned (for review see ref. 3). For example, an important series of studies among benzene-exposed subjects identified several differentially expressed genes in blood leukocytes which were related to immune function and leukemogenesis, an established outcome of benzene exposure^4^. However, the potential of blood-based omic profiles to reflect the impact of toxic exposures on cell function and associated disease pathogenesis processes in solid tissues is less well understood.

Exposure to tobacco smoke is one of the best studied examples of a common exposure with proven causal association with a variety of human diseases^5^,^6^. On this basis it provides an opportunity for the evaluation of the potential of blood-based omics (including cross-omics) profiling to reveal changes of relevance to exposure-related diseases and hence to predict corresponding disease hazards^1^,^7^. The impact of tobacco smoking on gene expression and CpG methylation profiles in blood leukocytes has been examined in a number of recent studies (see for example refs 8, 9, 10, 11), which have reported partly overlapping lists of features which are altered in smokers. Most of these studies focused primarily on the identification of biomarkers of tobacco smoke exposure, although the association of the altered profiles with particular cellular processes and diseases was discussed in some. Here we report on the impact of tobacco smoking on transcriptomic (including miRNA) and epigenomic (DNA methylation) profiles in buffy coats of apparently healthy subjects drawn from the general population, focusing in particular on the assessment of the observed changes in relation to diseases known to be associated with tobacco smoke. For this purpose we have identified smoking-induced profile changes, characterized them in terms of their biological information content and conducted disease connectivity analysis to identify diseases with which they are associated.

## Results

### Smoking-induced omic profile changes

**Figure 1:**
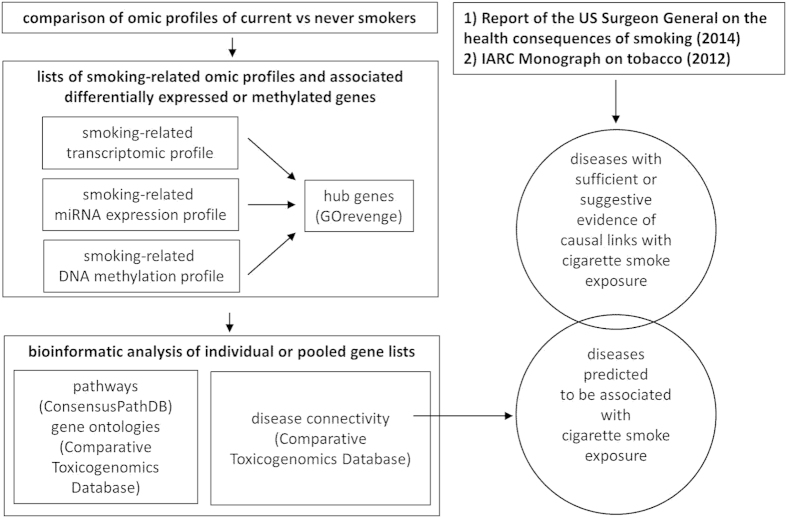
Flow of data and bioinformatics analyses. Further information on the bioinformatics tools employed is given in Methods.

We examined the impact of smoking on genome-wide gene expression and CpG methylation profiles in blood leukocytes of a total of 649 current, former and never smokers within two general population-based prospective cohorts, the Northern Sweden Health and Disease Study and EPIC Italy (Table 1). Our analysis of the resulting data and their relevance to tobacco-induced disease is diagrammatically outlined in Fig. 1.

Expression differed between current and never smokers for a total of 350 transcripts (FDR < 0.10; 231 FDR < 0.05) corresponding to 271 differentially expressed genes (DEGs) (information on cohort-stratified analyses is given in Supplementary Information). In agreement with previous studies^8^,^9^, we found most DEGs to be downregulated in current smokers and LRRN3 to be the most affected gene (upregulated) (Supplementary Table S1). No transcript showed a significant expression change in former smokers (smallest FDR > 0.99).

DNA methylation differed between current and never smokers at 1,273 CpG sites (FDR < 0.05; 184 at Bonferroni-corrected p < 0.05), the majority showing loss of methylation in current smokers (Supplementary Table S2). In agreement with previous studies^10^,^11^, we found the AHRR gene to be the most common epigenetic target, with 27 CpG sites significantly affected (FDR < 0.05). The affected CpG sites are associated with 725 differentially methylated genes (DMGs) or are located in intergenic regions. In former smokers, 17 CpG sites (FDR < 0.05; including 9 at Bonferroni-corrected p < 0.05) with reduced methylation relative to never smokers were observed, all of which were also significantly modified in current smokers (Supplementary Table S3).

Comparison of the miRNA profiles (only measured in 226 subjects from the Swedish cohort) showed 26 miRNAs to be overexpressed and 8 underexpressed in current smokers (FDR < 0.10; 8 at FDR < 0.05) (Supplementary Table S4). No significant change was observed in former smokers (smallest FDR > 0.99).

### Disease connectivity analysis

The Comparative Toxicogenomics Database^12^, which curates data describing relationships between chemicals, genes and diseases, was used to search for diseases related to the sets of DEGs and DMGs (individually or pooled) observed in current smokers and therefore predicted to be potentially associated with smoking. This search resulted in the identification of a total of 191 highly significant (Bonferroni-corrected p < 0.05) disease or condition MESH terms, presented in detail in Supplementary Table S5 and summarized in Table 2), comprising multiple disease categories. Addition to the above gene lists of the differentially expressed miRNAs had only a minor impact on the outcome (results not shown). On the other hand, as indicated in Supplementary Table S5 (last column), use of a subset of only 40 DEGs or DMGs, selected as described in the following section for their potential master-regulatory role (hub genes), predicted the majority of the above disease terms along with an additional 51 highly significant terms.

Table 2 shows that, for the great majority of the diseases or conditions predicted by the omic profiles, the epidemiological evidence of a causal association with tobacco smoking has been characterized as sufficient or, in a few cases, suggestive in the Report of the US Surgeon General on the health consequences of smoking^5^ or, for cancer, in the latest IARC Monograph on tobacco^6^. Notably this also holds for a few diseases (colitis, endometrial cancer) which show an inverse epidemiological association with smoking (decreased incidence in smokers). For a small number of predicted diseases, while no formal conclusion is given in the abovementioned major reports, evidence supportive of an association with smoking is mentioned therein (e.g. liver cirrhosis, Parkinson disease, demyelinating autoimmune disease such as multiple sclerosis) or they are well known to be associated with smoking-related diseases correctly predicted by omic profiling (e.g. cardiomegaly – a complication of heart disease; calcification of aortic valve – a precursor of aortic aneurism; liver cirrhosis – a late stage complication of liver fibrosis). Predicted diseases not discussed in the above reports, or for which the evidence of causal links with smoking is described as insufficient or clearly negative, include male genital, prostate and nerve tissue cancer, ventricular outflow obstruction, nephritis/glomerulonephritis, schizophrenia and disorders with psychotic features, lymphoma and adnexal disease. Finally, specific diseases or conditions for which there is sufficient evidence of an association with smoking but were not predicted by omic profiling include erectile dysfunction, reduced fertility in women and a number of diseases related to pulmonary infection (influenza, pneumonia, tuberculosis).

### Bioinformatics analyses

#### - Pathways associated with DEGs/DMGs

The mechanistic basis of the ability of DEGs and DMGs to predict smoking-related diseases was explored by conducting pathway analysis (ConsensusPathDB)^13^ using the two gene sets, separately as well as combined (totaling 894 unique genes). This resulted in 97 significantly (FDR < 0.05) overrepresented pathways, including multiple pathways related to TGF-β-, AhR- and NOTCH-signaling, blood coagulation, cell-cell and cell-matrix interactions, as well as pathways related to various diseases such as cancer and heart disease (Supplementary Table S6).

#### - Identification of hub DEGs/DMGs

To reduce the complexity of the list of DEGs and DMGs, we searched for genes with potential master-regulatory roles using GORevenge^14^, a bioinformatics tool that maps gene sets on to the hierarchical structure of the Gene Ontology graph tree and prioritizes them according to the number of GO terms to which they are linked. Fourty hub genes were thus identified which were linked to at least 30 (and upto 102) GO terms and included 12 DEGs and 30 DMGs, in their great majority underexpressed or/and undermethylated in smokers (Table 3). A large number of GO terms (derived using the Comparative Toxicogenomics Database) are highly significantly overrepresented in the set of hub genes, including multiple terms related to apoptosis, response to various endogenous and exogenous stimuli and protein metabolism (Supplementary Table S7). As already mentioned, disease connectivity analysis using these 40 hub genes resulted in the prediction of the majority of smoking-related diseases also predicted by the full sets of DEGs and DMGs (Supplementary Table S5).

**Figure 2:**
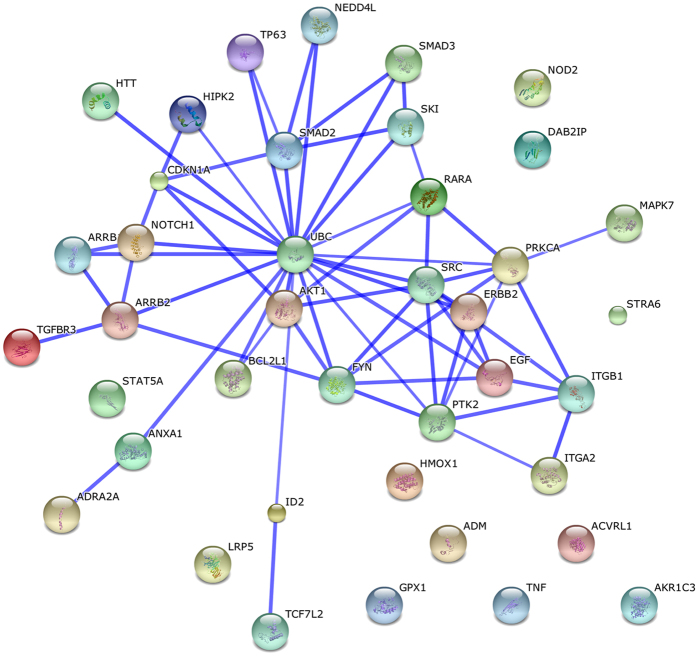
STRING-generated interaction network among the hub genes; the intensity of the edges reflects the strength of evidence; prediction methods: co-expression, experimentally observed interactions and curated databases; confidence score “high” (>70% probability of terms being found together in a metabolic map in the KEGG database).

To obtain a more global view of the organization of the smoking-related hub genes, we looked for networks of interactions between them using the online resource Search Tool for the Retrieval of Interacting Genes (STRING)^15^ which maps and integrates physical and functional protein-protein interactions. This revealed an extended network of interactions, including sub-networks centered on SMAD2 and SRC and tightly linked to a UBC node (Fig. 2).

#### - Hub genes in specific diseases

**Figure 3:**
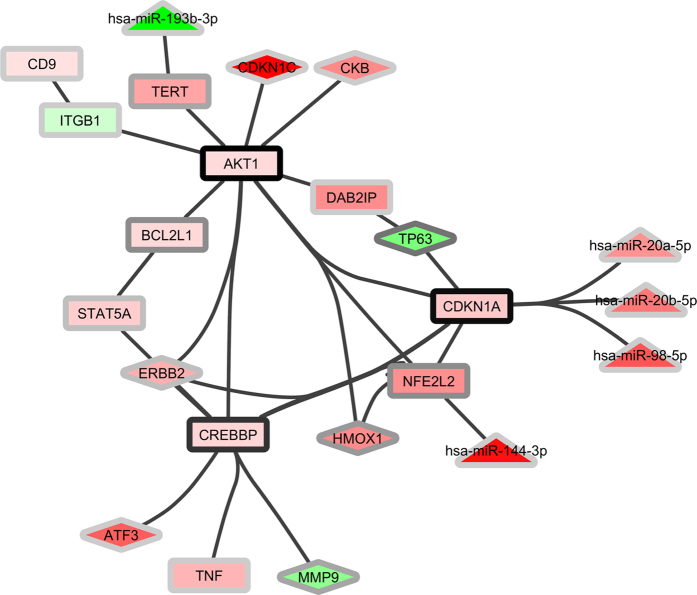
Interactions between DEGs, DMGs and miRNAs related to lung cancer as derived using ConsensusPathDB and Cytoscape; node shapes: diamonds = DEGs, rectangles = DMGs, triangles = miRNAs; node colours: red = down-regulated (the darker the more down-regulation), green = up-regulated (the darker the more up-regulation); the colours of the node borders indicate the number of connecting edges (the darker the more connecting edges).

We examined further the role of the hub genes in smoking-related diseases by first selecting those DEGs/DMGs identified by the Comparative Toxicogenomics Database as being associated with a specific disease (lung cancer) or with two disease categories (cancer and cardiovascular disease), all well known to be strongly linked to smoking (34, 178 and 105 DEGs/DMGs, respectively). Subsequent use of ConsensusPathDB and Cytoscape to identify and visualize the interactions between these genes showed (Fig. 3 and Supplementary Fig. S3) that hub genes AKT1 and CDKN1A serve as the main nodes linking multiple networks in all three cases, while SRC and PRKCA are additional major nodes in the two disease categories examined. Furthermore, the networks were extended with five DE-miRNAs to reveal that the differentially expressed miRNAs miR-20a-5p, miR-20b-5p and miR-98-5p are directly linked to the hub gene CDKN1A.

### Comparison of smoking-induced omic profiles with disease profiles

The ability of the DEGs and DMGs to predict smoking-related diseases results from the fact that these gene sets overlap significantly with lists of genes known independently to be linked with these diseases. Because our disease-predictive profiles reflect perturbations caused by tobacco smoke in apparently healthy smokers, the presence in them of genes also differentially altered in patients with smoking-related disease would provide a possible basis for linking exposure with early steps of disease pathogenesis. To explore this possibility, we went on to compare our smoking-related profiles with profiles reported to be differentially modified in blood cells of patients with two diseases strongly associated with smoking, namely lung cancer and coronary heart disease. In relation to lung cancer we used two published gene expression signatures observed in patients with non−small cell lung cancer^16^ and stage I lung adenocarcinoma^17^. In these studies, RNA extracted from whole blood was used to establish gene expression profiles optimally distinguishing between cases and controls. In both studies, the differential expression profiles had been derived while controlling for smoking status at the time of sampling and are therefore unlikely to include signals directly caused by recent exposure to tobacco smoke. As shown in Table 4, among 427 genes reported in the first study as being differentially expressed in subjects with lung cancer are included 11 of our smoking-related DEGs and 18 DMGs, including hub genes ADM and SMAD3. The probabilities, based on the hypergeometric distribution test, of a chance overlap of this magnitude are p = 0.024 and p = 0.25, respectively. In the case of the second study, which reported 49 differentially expressed genes, the corresponding overlaps are 5 DEGs (p = 4.22 × 10^−3^), including hub gene TGFBR3, and 5 DMGs (p = 0.028).

For the corresponding analysis of coronary heart disease we used the data from the most recent and largest published study^18^, in which RNA from total blood of subjects with or without disease was used to derive differential expression profiles. Comparison of a list of 592 unique genes which, after controlling for smoking status, were reported to be differentially expressed in subjects with disease (Supplementary Table 6 in ref. 18) with our lists of DEGs/DMGs showed an overlap of 21 DEGs (p = 3.3 × 10^−5^), including hub genes NEDD4L and TGFBR3, and 27 DMGs (p = 0.10), including hub genes BCL2L1 and CDKN1A. Furthermore, comparison with a list of 59 genes highlighted in the same report as having been found significantly associated with the coronary heart disease case/control status in this and 4 other comparable studies (Supplementary Table 3 in ref. 18), showed an overlap of 4 DEGs (p = 7.19 × 10^−3^) including hub gene NEDD4L and 3 DMGs (p = 0.34).

The above observations demonstrate that genes (including genes with major regulatory roles) differentially expressed in blood leukocytes of subjects with diseases causally associated with tobacco smoke exposure are found with high statistical significance to be also differentially expressed in smokers without these diseases. Analogous overlaps were also found, albeit at lower statistical significance, for genes with smoking-induced CpG methylation changes.

## Discussion

We agnostically examined in blood leukocytes of apparently healthy subjects the effects of tobacco smoke exposure on multiple types of genome-wide (omic) profiles and their association with disease. Given the extensive amount of independent epidemiologic knowledge available regarding the health effects of tobacco, our analysis serves as a proof-of-principle evaluation of the utility of blood-based omic profiles in relation to the identification of health hazards potentially associated with exposure to environmental and other toxic agents. In addition, as regards the health effects of smoking per se, the use of omic profiling as in the present study may provide new evidence for diseases not previously linked with smoking, as well as support for the identification of individuals with high susceptibility to tobacco-associated diseases.

**Figure 4:**
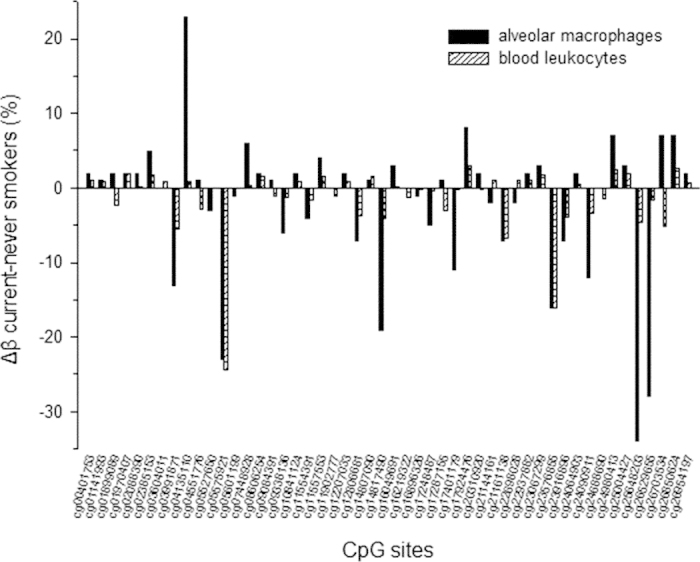
Comparison of smoking effects on the methylation of 49 AHRR CpG sites in blood leukocytes (this study) and lung alveolar macrophages^19^ at which the effects of smoking are significant (FDR < 0.05) in either tissue.

In agreement with many previous studies we identified LRRN3 and AHRR as the top expression and methylation gene targets, respectively (Supplementary Tables S1 and S2). The AHRR gene has been previously reported to be upregulated as well as epigenetically modified in the lungs of smokers^10^,^19^. We found that AHRR expression was also upregulated in blood leukocytes of smokers despite its very low basal level in this tissue relative to the lung^20^ (Supplementary Table S1). To take this comparison further we compared the changes we observed in CpG methylation in blood leukocytes to those previously reported for lung alveolar macrophages^19^. As shown in Fig. 4, of the 27 AHRR-associated CpG sites significantly (FDR  < 0.05) modified by smoking in blood leukocytes, 17 overlap with 39 sites reported to be modified (FDR  < 0.05) in alveolar macrophages (p for chance overlap = 1.31 × 10^−5^). Furthermore, the methylation changes at the 49 CpG sites modified by smoking in either tissue are highly correlated (Pearson r = 0.59; p < 10^−5^). While these observations relate to just one gene, they imply that DNA methylation changes under the influence of an external exposure may be qualitatively similar across tissues regardless of the tissue-specific basal expression levels, thus providing a biological justification for the use of blood-based CpG methylation data in the prediction of effects in other tissues.

### Smoking-induced blood omic profiles and disease prediction

We conducted disease connectivity mapping using as input the DEG/DMG lists obtained with the pooled cohort dataset, rather than the smaller lists of genes replicating between the two cohorts, having in mind the significant overlap of the former with previous reports^8^,^9^,^10^,^11^. In deriving these lists we adjusted for cohort, age, sex and BMI (additional adjustment for education, physical activity and alcohol consumption had only a minor impact). While the possibility that additional parameters might have confounded these lists cannot be completely excluded, it is highly unlikely that other confounders (i.e. exposures that both modify the expression/methylation profiles and are associated with tobacco smoking) are relevant.

The combination of DEGs and DMGs shows a remarkable ability to predict almost all diseases or conditions for which there is sufficient or suggestive epidemiological evidence of a causal link with smoking, as well as diseases or conditions known to be closely associated, as precursors or late-stage complications, with such diseases (Table 2 and Supplementary Table S5). The set of DMGs alone was able to correctly predict a large fraction of smoking-related diseases despite the fact that only few of these genes had their expression also altered by smoking, reinforcing the suggestion that exposure-induced epigenetic changes in blood cells may extend to additional tissues and contribute to the initiation and progression of disease therein.

For a number of omics-based disease predictions, the epidemiological evidence is negative or too limited to support a causal link with tobacco smoking^5^,^6^, meaning that these diseases may represent false positive predictions (male genital, prostate and nerve tissue cancer, lymphoma, ventricular outflow obstruction, nephritis/glomerulonephritis, adnexal disease, schizophrenia and disorders with psychotic features). While for lymphoma there is some epidemiological evidence of links with smoking^21^,^22^, most of the remaining possible false positives may reflect the fact that they are linked to, and share genes with, other conditions known to be caused by smoking. This is supported by the results of hierarchical clustering analysis of the associations between diseases and smoking-modified genes (Supplementary Fig. S4) which shows that, for example, nephritis-related conditions and adnexal diseases cluster close to immune system-related and ovarian diseases, respectively, both known to be caused by smoking.

The identification of schizophrenia and disorders with psychotic features as a disease category potentially associated with tobacco smoking is of particular interest. This could possibly reflect an inverse causation effect, i.e. subjects with undiagnosed disease tending to smoke more as a result of nicotine-induced release of dopamine leading to relief of symptoms^5^ (see relevant note in Table 2). In this context it is notable that in Supplementary Fig. S4 schizophrenia clusters with substance-related disorders. On the other hand, a series of recent reports from large epidemiological studies, including prospective cohort studies and meta-analyses, consistently suggests the possibility of a positive causal association between tobacco smoking and this disease category^23^,^24^,^25^. Seen in the latter context, our finding highlights the potential of omic profiling to provide independent molecular evidence in support of weak epidemiological observations.

Finally, a small number of specific diseases for which the evidence of a causal association with smoking has been characterized as sufficient was not predicted by omic profiling and therefore they may be considered as false negatives. These diseases include erectile dysfunction, reduced female fertility as well as a number of conditions related to pulmonary infection (influenza, pneumonia, tuberculosis). For the first of these diseases the most common cause is arteriosclerosis, which is correctly predicted to be associated with smoking, and a likely explanation for the failure to predict it may be related to the fact that only 9 genes are currently linked by the Comparative Toxicogenomics Database to this disease. As regards reduced female fertility, it is possible that this condition may result from the known ability of cigarette smoke to cause increased follicle death and altered hormone output^26^, conditions which may be reflected in the prediction of ovarian diseases. Finally, while pulmonary infection-related diseases were not predicted by our omic profiling analysis, it is notable that use of the list of hub genes derived without adjustment of the epigenetic profiles for the variation in white blood cell sub-populations, and hence reflecting changes in the proportions of immune cells, resulted in the prediction of most disease terms described in Supplementary Table S5 and, in addition, of multiple terms related to bacterial and viral infection (results not shown).

The partly overlapping nature of many of the health conditions involved precludes a proper quantitation of the predictive ability of omics profiling. However an indicative estimate can be obtained by taking as the total number of predictions the conditions represented by the number of rows of Table 2 (59), of which, according to the preceding discussion, 8 may be considered as false positives (including lymphoma and schizophrenia in accordance with the conclusions of the two major reference evaluations employed^5^,^6^) and 51 as correctly predicted. By subtracting from this number the 8 false positives and adding the 3 false negatives discussed above, the total number of “true” smoking-related conditions can then be estimated as 54. This leads to indicative estimates of sensitivity (=correctly predicted/“true”) and positive predictive value (=correct predictions/all predictions) of omics profiling-based prediction of 94% and 86%, respectively (specificity cannot be estimated). While these estimates are only indicative, they provide strong support to the conclusion that omics profiling is remarkably reliable in predicting smoking-related health conditions.

### Effects of smoking on biological pathways

Pathway analysis (Supplementary Table S6) using the DEG and DMG sets provides mechanistic support for the potential exposure-effect associations identified. This is illustrated in Supplementary Table S8 which summarizes the changes in component genes of 6 pathways with central role in smoking-related diseases and shows that nearly 1 in 3 of the genes involved was among those found in our study to be modified in smokers.

### Smoking-modified hub genes

Among the genes significantly modified in smokers we identified 40 hub genes (Table 3) which play a central regulatory role in the cellular changes induced by smoking and can predict most tobacco-related diseases (Supplementary Table S5). The key role of these hub genes in the cellular perturbations caused by smoking is illustrated in Fig. 3 and Supplementary Fig. S3, where the interactions between DMGs/DEGs/DE-miRNAs associated with lung cancer or the disease categories cancer and cardiovascular diseases, respectively, are shown. In all three cases, two hub genes (AKT1 and CDKN1A) function as nodes linking multiple interacting networks, both also being known to play an important role in these diseases and to be modulated by cigarette smoke exposure^27^,^28^,^29^. Strikingly, CDKN1A, which was significantly undermethylated at 4 CpG sites in smokers (Table 3), is targeted by 3 DE-miRNAs (Fig. 3 and Supplementary Fig. S3). In the case of the two disease categories cancer and cardiovascular disease, in addition to AKT1 and CDKN1A, central regulatory roles appear to be played by hub genes SRC and PRKCA which are also known to be related to the diseases and modulated by cigarette smoke exposure^30^,^31^,^32^,^33^,^34^. While we did not detect a significant effect of smoking on the expression of these genes in blood leukocytes, CpG methylation was significantly affected in all cases.

The smoking-modified hub genes appear to be organized primarily around two sub-networks of major importance in multiple cellular functions, namely TGF-β (centred on SMAD2) and EGFR/ERBB2 (centred on the EGFR-SRC axis) signaling, which are in turn highly linked to a UBC node (Fig. 2). The UBC gene, which codes for a polyubiquitin precursor, was significant undermethylated in smokers. Furthermore it was represented in our transcriptomics dataset by 4 expression probes, all of which were underexpressed in smokers (average decrease 3.41%). While this change was not statistically significant, it suggests a probable downregulation of expression of the gene. The central location of UBC in the network of interactions between the hub genes suggests that alterations in ubiquitination may mediate many of the cellular effects of smoking, in line with the evidence described in the preceding paragraph in relation to cancer and cardiovascular diseases. Ubiquitination is intimately related to protein catabolism via the ubiquitin-proteasome system, but it can also affect cellular processes by altering the activity of proteins. In addition, free polyubiquitin has distinct roles in the activation of protein kinases and in signaling^35^. Existing evidence indicates that changes in the ubiquitin system play an important role in the development of smoking-related diseases including cancer, cardiovascular, neurodegenerative, respiratory and immune system diseases^36^. Our suggestion of a central role of ubiquitination in cellular signaling changes in smokers is in line with reports that the ubiquitination pathway protects cells from the detrimental effects of proteins damaged by exposure to cigarette smoke^37^ and that the protein ubiquitination pathway is one of the top pathways perturbed in human peripheral blood mononuclear cells treated *in vitro* with cigarette smoke extract^38^.

The above discussion indicates that hub genes altered in blood cells of apparently healthy smokers play important roles during the pathogenesis of smoking-related diseases in target tissues other than blood. This in turn suggests that such hub genes form part of adverse outcome pathways^1^ which constitute early events in disease pathogenesis and may therefore be particularly appropriate candidate «meet-in-the-middle» biomarkers linking toxic exposures to related diseases and potential tools for use in risk assessment^7^. This suggestion is supported by the fact that hub genes form part of the highly significant overlaps between our smoking-induced gene expression profiles and profiles observed in patients with two diseases strongly associated with smoking, namely lung cancer and coronary heart disease (Table 4). These overlaps include hub genes ADM and TGFBR3, the second of which was observed in relation to gene expression profiles in lung cancer^17^ and coronary heart disease^18^ patients. TGFBR3 is known to behave as a suppressor of the progression of multiple types of cancer including lung cancer^39^, which is in harmony with our observation that its expression is significantly reduced in smokers (Table 3). Other smoking-related hub genes found to overlap with coronary heart disease-related expression profile include NEDD4L, BCL2L1 and CDKN1A. A genetic variant of NEDD4L has been reported to be associated with postural change in systolic blood pressure, a risk factor for cardiovascular and coronary heart disease^40^. It is also notable that the overlap also includes LRRN3, top target for smoking in relation to gene expression (overexpressed) in blood leukocytes. We have also found that 2 CpG sites associated with LRRN3 suffer significant (FDR < 0.05) loss of methylation in smokers (result not shown). LRRN3 appears to be involved mainly in neurodevelopment and its possible role to heart disease is currently unclear. Nevertheless, its presence in the differential gene expression profiles of patients with coronary heart disease suggests that it may serve as a marker linking past tobacco smoke exposure with disease.

### Concluding remarks

Our study demonstrates that the combination of changes in gene expression (including miRNA) and CpG methylation in blood leukocytes of smokers is able to predict with high sensitivity and specificity diseases which occur in tissues other than blood or hematopoietic tissues and whose incidence is affected by tobacco smoking. We have also shown that there is a close similarity in the effects of cigarette smoke on the expression and CpG methylation of the AHRR gene in blood leukocytes and the lung of smokers. Taken together, these observations are compatible with the reported operation of common gene regulation networks across different tissues which are more highly connected than within-tissue networks^41^,^42^. They also suggest that blood cells may respond to toxic exposures in a manner similar to solid tissues, thus extending to early steps of disease pathogenesis the implications of the reported observation that the molecular signature of a given disease tends to be robust across different tissues and more prominent than the signature of each tissue or other influences^43^. Finally, an additional factor which may contribute to the concordance of early disease signatures in blood and other tissues relates to the possibility that the genomic profiles of blood cells reflect interactions with metabolites or signaling molecules released by solid tissues. The overall outcome of the combined operation of these mechanisms is that blood-based omic profiles of environmental or other types of toxic exposure may also contain signatures of early disease-related perturbations occurring in distant tissues and therefore be useful in the derivation of intermediate biomarkers which reflect etiological exposure-disease links.

## Methods

The study was conducted in the context of the European EnviroGenomarkers project (www.envirogenomarkers.net) and involved subjects from the European Prospective Investigation into Cancer and Nutrition study (EPIC-ITALY) and the Northern Sweden Health and Disease Study (NSHDS) (Table 1)^44^,^45^. Both studies used population-based recruitment with standardized lifestyle (including smoking) and personal history questionnaires, anthropometric data and blood samples collected at recruitment (1993–1998 for EPIC-ITALY; 1990–2006 for NSHDS). Subjects were categorized as current, former or never smokers on the basis of their declared smoking status at the time of recruitment. The duration of smoking for current smokers ranged 2.1–46.5 years (mean 31.0 years) years while time since quitting for former smokers ranged 4 months to 38 years (mean 14.3 years), with no significant dfferences between the two cohorts. Data on smoking intensity (pack-years) were available only for the EPIC Italy cohort.

The EnviroGenomarkers project was originally designed as two nested case-control studies, one for B-cell lymphoma and one for breast cancer. The participants included 93 incident cases with breast cancer, 229 incident cases of B-cell lymphoma and 327 controls. No participant was diagnosed with disease within less than 2 years of blood sample collection and for this reason all participants were treated as apparently healthy at recruitment. In order to minimize effects on the omic profiles, subjects were included in the current study only if the processing of their blood samples had been completed within 2 hours of collection^46^. The EnviroGenomarkers project and its associated studies and protocols were approved by the Regional Ethical Review Board of the Umea Division of Medical Research, as regards the Swedish cohort, and the Florence Health Unit Local Ethical Committee, as regards the Italian cohort, and all participants gave written informed consent. The studies were conducted in accordance with the approved guidelines.

### Analytical procedures and data processing

RNA and DNA extraction from buffy coats, genome-wide analysis of gene expression (Agilent 4 × 44K human whole genome microarray platform) and CpG methylation (Illumina Infinium HumanMethylation450 platform), miRNA expression profiling [Agilent Human miRNA Microarray (Release 19.0, 8 × 60K), representing 2006 human miRNAs], and the corresponding data quality assessment and preprocessing, were conducted as described previously^46^,^47^. Missing values imputation was applied (k-nearest neighbor). Methylation data, expressed as M-values corresponding to the logarithmic ratio of the methylated versus the unmethylated signal intensities, were preprocessed initially with GenomeStudio (version 2011.1) Methylation module (version 1.9; Illumina). Subsequently, data normalization to address the issue of unwanted technical variation was performed, using scripts written and run in MATLAB environment (Mathworks, Release 2012b), by making use of the DNA methylation measured in multiple replicates of a technical control sample randomly distributed among the study samples as previously described^48^ by a procedure involving two successive steps of intensity-based correction (a) within-chip and b) across all probes). Probes giving mean methylation for all samples in the range 0%–4% or 96–100% were omitted from further analyses. From the resulting subset, 410,987 probes targeting autosomal CpG loci in the 22 chromosomes (sex chromosomes excluded) were selected for statistical analysis. CpG loci containing SNPs at a distance less than 3 nucleotides from the corresponding cytosine and minor allele frequency >10% were omitted from the significant CpG hit lists.

### Statistical analyses

Linear mixed models were used for all statistical analyses, using M values for DNA methylation or log_2_intensities of mRNA or miRNA expression as dependent variables, with the Array Studio software package (Omicsoft, Cary, NC, USA, version 8.0.1.32) and R software (version 3.0.2). Date of isolation, labeling, and hybridization for RNA expression, date of analysis for methylation and batch number for miRNA expression were also included in the models as random variables to account for technical variation. All analyses additionally adjusted for sex, age, BMI and cohort (the inclusion, in addition, of education, physical activity and alcohol consumption was also checked but rejected as having a minor impact). Owing to the design of the EnviroGenomarkers project, described above, future disease (breast cancer, B-cell lymphoma) and case-control status were also included as fixed variables. Inclusion of these incident cases did not appear to bias the resulting lists of smoking-related signals (see Supplementary Information). In the case of DNA methylation data, the models were also adjusted for blood cell composition estimated using the algorithm developed by Houseman *et al*.^49^. For this purpose, cell count predictions were obtained using cell-sorted DNA methylation data, publicly available in the *FlowSorted.Blood.450k* Bioconductor package, as previously described^50^. Multiple testing was accounted for with high stringency by using Bonferroni or FDR Benjamini-Hochberg correction.

### Selection of hub genes using GoRevenge

In order to select genes with potential master-regulatory roles, the list of DEGs and DMGs was submitted to GORevenge^14^, a web application that maps gene sets on to the Gene Ontology graph tree, exploiting its hierarchical structure in order to assess their functional relevance but at the same time efficiently corrects for semantic annotation bias resulting from the unbalanced level of description of the various cellular processes. This is done through the use of appropriate graph-based semantic similarity measures which finally let the tool yield a sorted list of genes prioritized according to the final number of GO terms to which they are linked.

### Disease connectivity analysis

Diseases or conditions enriched with smoking-modified genes were identified using the “set analyzer” tool of the Comparative Toxicogenomic Database (http://ctdbase.org) which provides manually curated information about chemical-gene/protein interactions, chemical-disease and gene-disease relationships. The lists of genes (DEGs, DMGs, DE-miRNAs, hub genes) were introduced into tool (gene names not recognized were replaced by synonyms selected from GeneCards) and the returned list of enriched diseases with Bonferoni-corrected p < 0.05 collected.

### Pathway analysis and visualisation

Pathways associated with the combined sets of DEGs and DMGs was performed by an “over-representation analysis” in ConsensuspathDB^13^ (Release 30) using standard parameters and FDR < 0.05. A background list consisting of all genes measured (either transcriptomic or epigenomic) was used in the analysis and the default pathway selection option consisted of a minimum overlap of 2 genes with the input list of DEGs and/or DMGs. Furthermore, DEGs, DMGs and DE-miRs identified by the Comparative Toxicogenomics Database as being associated with a specific disease or disease category were subjected to an “induced network module” analysis also provided by ConsensuspathDB. The induced networks thus obtained were exported to Cytoscape (v3.2.0) where, using the CyTargetLinker plugin (v3.0.1)^51^, validated microRNA-gene interactions (based on the regulatory interaction networks of DE-miRs identified by means of miRTarBase release 4.4) were obtained. Multiple edges between nodes were bundled and self-loops were removed. Finally, the expression changes of the DEGs, DMGs and DE-miRs were visualised on the gene-gene/gene-miRNA interactions network.

## Additional Information

**How to cite this article**: Georgiadis, P. *et al*. Omics for prediction of environmental health effects: Blood leukocyte-based cross-omic profiling reliably predicts diseases associated with tobacco smoking. *Sci. Rep*. **6**, 20544; doi: 10.1038/srep20544 (2016).

## Supplementary Material

Supplementary Information

## Figures and Tables

**Table 1:** Population study data.

		**Total population**	**EPIC Italy**	**NSHDS**
N	total	649	250 (38.5% of total)	399 (61.5% of total)
	male	236 (36.4% of total)	65 (26.0% of EPIC Italy)	171 (42.9% of NSHDS)
	female	413 (63.6% of total)	185 (74.0% of EPIC Italy)	228 (57.1% of NSHDS)
age; mean (SD)		52.1 (7.8)	53.3 (8.1)	51.4 (7.6)
BMI; mean (SD)		25.8 (3.9)	25.8 (3.5)	25.8 (4.1)
smoking status, N	current smokers	143 (22.0% of total)	61 (24.4% of EPIC Italy)	82 (20.6% of NSHDS)
	never smokers	311 (47.9% of total)	120 (48.0% of EPIC Italy)	191 (47.9% of NSHDS)
	former smokers	195 (30.0% of total)	69 (27.6% of EPIC Italy)	126 (31.6% of NSHDS)
duration of smoking	N*	130	60	70
smoking intensity (pack-years)#	years (SD)	31.0 (15.2)	29.5 (7.0)	32.2 (9.6)
	N*	59	59	
	pack-years (SD)		410.3 (323.1)	–

^*^number of subjects for whom information was available; # available only for EPIC Italy.

**Table 2:** Summary of diseases predicted by cross-omics profiling and the Comparative Toxicogenomics Database (Bonferoni-corrected p < 0.05; full list shown in Supplementary Table S5) and comparison with the conclusions of the US Surgeon General’s Report^5^ and the IARC Monograph on tobacco^6^; disease-related terms from Supplementary Table S5 have been grouped by disease category after excluding generic categories and repetitions.

Disease categories	Disease/condition name	US Surgeon General’s Report^5^		Comments
		Formal conclusion(s) regarding evidence of causal relationship with smoking	Other relevant remarks	
cancer	acute myeloid leukemia	sufficient		IARC^6^, sufficient evidence
	breast cancer	suggestive		IARC, positive association
	bronchogenic carcinoma	sufficient		IARC, sufficient evidence
	colorectal cancer	sufficient		IARC, sufficient evidence
	esophageal cancer	sufficient		IARC, sufficient evidence
	gastrointestinal cancer	sufficient (stomach, colorectum)		IARC, sufficient evidence (stomach, colorectum)
	genital cancer, female	sufficient (cervix); sufficient (reduction of endometrial cancer risk in post-menopausal women)		IARC, sufficient evidence (cervix, ovary)
	genital cancer, male	not discussed		IARC, no consistent association
	germ cell neoplasms	inadequate (ovarian cancer)		IARC, sufficient evidence (ovary)
	head and neck neoplasms	sufficient (oral cavity, pharynx, larynx)		IARC, sufficient (oral cavity, naso-, oro-, hypo-pharynx, nasal cavity and accessory sinuses, larynx)
	liver cancer	sufficient		IARC, sufficient evidence
	lung cancer	sufficient		IARC, sufficient evidence
	lymphoma	not discussed		IARC, evidence inconclusive; IARC, positive association between parental smoking and childhood acute lymphocytic leukemia; limited evidence of association with risk of Hodgkin and non-Hodgkin lymphoma^21^,^22^
	nerve tissue neoplasms	suggestive of no causal link (brain)		IARC, evidence inconclusive
	pancreatic neoplasms; endocrine gland cancer	sufficient (pancreas)		IARC, sufficient evidence (pancreas)
	prostate cancer	suggestive of no causal relationship		IARC, no consistent association
	urinary bladder cancer	sufficient		IARC, sufficient evidence
	urogenital neoplasms	sufficient (renal cell; renal pelvis; urinary bladder); sufficient (cervix); sufficient (reduction of endometrial cancer risk in post-menopausal women)		IARC, sufficient evidence (cervix, ovary, kidney, ureter, urinary bladder; inverse association with endometrial cancer risk)
	uterus	sufficient (cervix)		IARC, sufficient evidence (cervix)
cardiovascular diseases and related conditions	aortic aneurysm; calcification of aortic valve	sufficient (abdominal aortic aneurysm);		calcification of aortic valve is associated with aortic aneurysm
	arterial occlusive diseases; coronary artery disease; arteriosclerosis; reperfusion injury	sufficient (subclinical atherosclerosis)		reperfusion injury is caused when blood supply returns to a tissue after ischemia; associated with microvascular dysfunction
	cerebrovascular disorders	sufficient (stroke)		
	embolism and thrombosis; blood coagulation disorders; hypertension	sufficient (stroke; cardiovascular disease)	p. 430: evidence that exposure to secondhand smoke may increase the risk of hypertension	positive association of smoking with higher risk of mortality from hypertensive heart disease^52^
	myocardial ischemia; myocardial infarction; ventricular remodeling; cardiomegaly	sufficient (coronary heart disease, heart failure)		
congenital abnormalities and related conditions	cardiovascular abnormalities	suggestive (atrial septal heart defects)		
	craniofacial abnormalities; mucoskeletal abnormalities	sufficient (smoking in early pregnancy and orofacial clefts); suggestive (other types of abnormalities)		
	ventricular outflow obstruction	not discussed	p. 476: reports of association between maternal smoking and outflow tract defects	limited supportive evidence^53^
connective tissue disease	rheumatoid arthritis	sufficient		
digestive system disease	colitis	suggestive (reduction of risk)		
	Crohn’s disease	sufficient		
	gastroenteritis	not discussed	p. 62: sufficient evidence that smoking compromises immune homeostasis; smoking is a determinant of the incidence of a large number of diseases related to immunologic dysregulation, including diverse viral and bacterial infections, especially but not exclusively of the lungs	
	liver cirrhosis	not discussed	p. 569: smoking is a risk factor for liver fibrosis	cirrhosis is a late stage of liver fibrosis
	rectal diseases	sufficient (colorectal cancer)		IARC, sufficient evidence (rectal cancer)
	stomach diseases	sufficient (stomach cancer; gastric ulcer in persons who are Helicobacter pylori positive)		
endocrine system disease; metabolic disease	diabetes mellitus, type 2; glucose metabolism disorders, hyperinsulinism	sufficient (diabetes type 2)		
	ovarian diseases	not discussed	sufficient evidence of reduced female fertility	evidence of increase in follicle death and altered hormone output^26^; IARC, sufficient evidence (ovarian cancer)
eye disease	eye diseases	sufficient (neovascular and atrophic forms of age-related macular degeneration; cataract)		
immune system disease and related conditions	asthma; respiratory hypersensitivity; berylliosis	suggestive (asthma); suggestive (nonspecific bronchial hyperresponsiveness)		berylliosis is a chronic allergic-type lung disease with symptoms overlapping with those of asthma
	autoimmune diseases; calcinosis	sufficient (rheumatoid arthritis); suggestive (Crohn’s disease)		calcinosis is associated with autoimmune diseases, e.g. rheumatic arthritis^54^
	demyelinating autoimmune diseases, CNS; neuromuscular diseases; gliosis	not discussed	p. 569: smoking is a risk factor for multiple sclerosis; sufficient evidence of causal links of smoking with compromised immune homeostasis and altered immunity associated with an increased risk for several disorders with an underlying immune diathesis	astrogliosis is associated with neuroinflammatory disorders^55^
	immunoproliferative disorders; lymphoproliferative disorders	not discussed		limited evidence of association with risk of lymphoma^21^,^22^; IARC, positive association between parental smoking and childhood acute lymphocytic leukaemia
mental disorder; nervous system disease; brain diseases	mental disorders diagnosed in childhood	suggestive (maternal prenatal smoking and disruptive behavioral disorders, and attention deficit hyperactivity disorder, in particular among children)	sufficient evidence that nicotine exposure during fetal development has lasting adverse consequences for brain development	
	schizophrenia and disorders with psychotic features	insufficient to infer the presence or absence of a causal relationship between maternal prenatal smoking and schizophrenia in her offspring	p. 124: nicotine-induced release of dopamine could improve attention and processing symptoms and sensory-gating deficits in schizophrenia	evidence of positive, causal association of smoking with risk of schizophrenia^23^,^24^,^25^
	substance-related disorders; neurotoxicity syndrome; heavy metal poisoning	sufficient (nicotine-addiction and related conditions)		
	epilepsy	not discussed		evidence of positive association of smoking with risks of epileptic seizure^56^
	hyperalgesia; pain; somatosensory disorders	not discussed		evidence of altered pain sensation in smokers^57^
	Parkinson disease; basal ganglia disease; movement disorders; manganese poisoning		p. 123: evidence of protective effect (Parkinson disease)	manganese poisoning is associated with increased risk of Parkinson disease^58^
mouth disease	stomatognathic diseases	sufficient (periodontitis); suggestive (dental caries)		
musculoskeletal disease	osteoporosis; calcium metabolism disorders	sufficient (osteoporosis)		
	psoriatic arthritis	not discussed		complication of psoriasis; evidence of positive association of smoking with psoriasis^59^
pathology (anatomical condition)	hypertrophy; hyperplasia	not discussed		associated with heart disease (hypertrophy) and cancer (hyperplasia)
respiratory tract disease and related conditions	obstructive lung diseases; bronchial diseases; fibrosis;	sufficient (COPD); suggestive (idopathic pulmonary fibrosis); sufficient (all major respiratory symptoms among adults, including coughing, phlegm, wheezing and dyspnea)		
signs and symptoms	overweight; obesity	sufficient (maternal active smoking and fetal growth restriction and low birth weight)		smoking is independently associated with an increased risk of central obesity and lower BMI^60^
urogenital disease	female urogenital diseases and pregnancy complications	sufficient (ectopic pregnancy, premature rupture of the membranes, placenta previa, placental abruption, preterm delivery and shortened gestation, maternal active smoking and fetal growth restriction and low birth weight; reduced risk for preeclampsia); suggestive (spontaneous abortion)		
	nephritis/glomerulonephritis IGA	not discussed	p. 569: smoking is a risk factor for multiple sclerosis; sufficient evidence of causal links of smoking with compromised immune homeostasis and altered immunity associated with an increased risk for several disorders with an underlying immune diathesis	associated with immune system malfunction;
	kidney disease	sufficient (kidney cancer)		IARC, sufficient evidence (kidney cancer)
	adnexal diseases	not discussed		no supportive evidence
pathology (process)	postoperative complications	sufficient (adverse surgical outcomes related to wound healing and respiratory complications)		
	hemorrhage		pp. 419, 423: associated with stroke, intracerebral hemorrhage, ischemia, thrombosis (sufficient evidence)	

**Table 3:** Hub genes: DEGs and DMGs associated with 30 or more GO terms as derived in GoRevenge; sorted by decreasing no. of links to GO terms; when fold change data from multiple expression probes related to the same gene were available, the mean value is shown; settings: Distance = graph, Relaxation = 0.

Gene	No. of links to GO terms	Fold change, expression	Change in methylation of affected CpG sites (%)
NOTCH1	102		−1.36
TNF	93		−1.32; −0.93; −0.89; −0.71; −0.65; −0.62; −0.37
AKT1	90		−1.31
SMAD3	69		−1.81
NOD2	67		−1.75; −1.02
UBC	61		−1.87
DAB2IP	59		−0.22
PRKCA	52		−1.48
ITGB1	50		1.37
TCF7L2	49	0.83	
RARA	46		−5.01; −1.60; −1.36; −0.93; 1.29
STAT5A	46		−0.92; −0.51
PTK2	45	0.85	−3.16
GPX1	45		−1.51
TP63	44	1.26	
SRC	42		1.27; 1.29
LRP5	41		−3.01; −2.32; −1.40; −1.03; −0.90
HTT	41		−1.98
ADM	40	1.30	
SMAD2	39		−0.84
BCL2L1	39		−1.38
HMOX1	39	0.84	
ID2	39	0.85	
CDKN1A	38		−2.42; −1.82; −1.28; −0.74
ITGA2	38		−1.71
ADRA2A	38	0.82	−3.25
ARRB2	37		−0.36
SKI	36		−1.43; −0.55; 3.16
ACVRL1	34		−0.92
STRA6	34		−1.78
ARRB1	34		−2.95
FYN	33		−0.60
HIPK2	33		−1.61
EGF	33		1.47
NEDD4L	33	1.18	
MAPK7	32	0.92	
ERBB2	31	0.89	
AKR1C3	31	0.81	
TGFBR3	31	0.83	
ANXA1	30		−0.87

**Table 4:** Overlap of smoking-related DEG/DMG profiles with reported differential expression profiles in blood leukocytes of patients with lung cancer or cardiovascular disease; bold characters indicate hub genes.

	Lung cancer		Cardiovascular disease	
	Zander *et al*.^16^	Rotunno *et al*.^17^	Joehannes *et al*.^18^	common to Joehannes *et al*.^18^ and other studies (Suppl. Table 6, ref. 18)
no. of genes differentially expressed in cases (disease profile)	427	49	592	59
smoking-related DEGs also found in disease profile	number: 11 (p = 0.024)* list: **ADM**, CEACAM1, DSC2, FEZ1, GPBAR1, IL2RB, LGR6, PLOD2, PPBP, RARRES3, SYT17	number: 5 (p = 4.22 × 10^−3^)* list: CYP1B1, F13A1, GZMB, RUNX3, **TGFBR3**	number: 21 (p = 3.4 × 10^−5^)* list: ACRBP, ARG2, C15orf26, C1orf21, CA2, CDK2AP1, CTSW, FGFBP2, GPR56, GZMA, GZMB, KLRF1, **NEDD4L**, NKG7, PRF1, RBX1, SAMD3, SLAMF7, SUCNR1, **TGFBR3**, XPNPEP1	number: 4 (p = 9.53 × 10^−3^)* list: LRRN3, **NEDD4L**, PDGFD, SLAMF7
smoking-related DMGs also found in disease profile	number: 18 (p = 0.25)* list: ABLIM1, CACHD1, CD58, CD96, CNTNAP2, E2F1, EWSR1, LSM4, MLL, MORC2, NFE2L2, NT5C2, PABPC4, PHF15, PHF19, S100P, **SMAD3**, UBE2C	number: 5 (p = 0.028)* list: AUTS2, CD96, GNB2L1, RUNX3, STAT4	number: 27 (p = 0.12)* list: ASAP1, BAMBI, **BCL2L1**, C13orf15, CD9, **CDKN1A**, EPB49, GPR56, HIST1H2BJ, HK1, HOMER2, JAZF1, LNX2, MKRN1, MYLK, PARD3, PHOSPHO1, RILP, RNF182, SGIP1, SH3BGRL3, SLC1A5, SLC24A3, ST3GAL1, STOML2, TFDP1, TTPAL	number: 3 (p = 0.38)* list: KIAA0319L, LRRN3, TFDP1

^*^hypergeometric distribution test p for over-representation of smoking-related hub genes among the genes reported to be differentially expressed in cases.
