# Supplementary material for: Omics for prediction of environmental health effects: Blood leukocyte-based cross-omic profiling reliably predicts diseases associated with tobacco smoking
Source: Sci Rep. 2016 Feb 3;6:20544. doi: 10.1038/srep20544 (PMC4738297; doi:10.1038/srep20544)
Supplement: Supplementary Information [file srep20544-s1.pdf]

SUPPLEMENTARY INFORMATION

Title: Omics for prediction of environmental health effects: Blood leukocyte-based cross-omic profiling reliably predicts diseases associated with tobacco smoking

Authors: Panagiotis Georgiadis, Dennie G. Hebels, Ioannis Valavanis, Irene Liampa, Ingvar A. Bergdahl, Anders Johansson, Domenico Palli, Marc Chadeau-Hyam, Aristotelis Chatziioannou, Danyel G.J. Jennen, Julian Krauskopf, Marlon J. Jetten, Jos C.S. Kleinjans, Paolo Vineis and Soterios A. Kyrtopoulos, on behalf of the EnviroGenomarkers consortium

Assessment of the robustness of the smoking-related omic changes across the cohorts

The robustness of the smoking-related omic profile changes identified using the full dataset was assessed in the two individual cohorts of the study. In these stratified analyses, for current smokers the total numbers of significant (FDR<0.10) transcripts were 184 and 114, respectively, with a statistically significant ($p<10^{-5}$) overlap of 6 transcripts (marked in Supplementary Table S1 online), while the corresponding numbers of total significant (Bonferoni p<0.05) epigenetic signals in the individual cohorts were 101 and 22, respectively, of which 21 were shared (marked in Supplementary Table S2 online). Of the 350 transcriptomic signals declared as statistically significant (FDR<0.10) in the full dataset, 135 and 18 are also significant at the same stringency in stratified analyses based on the Swedish and Italian cohorts, respectively. For epigenetic analyses, of the 184 signals declared as significant (Bonferoni-corrected p<0.05) in the full dataset, 84 and 22 were similarly significant in the Swedish and Italian cohorts, respectively.

Supplementary Fig. S1 shows the $-\log_{10}$p-values of the signals identified as significant in the full dataset, plotted against the p-based ranking in the latter dataset, where the overlaps between the individual and pooled cohorts are illustrated. It can be seen that, although the p-values are weaker in the individual cohorts (especially in the smaller, EPIC Italy cohort), their variation in all three datasets is similar.

The above observations suggest that the differences between the results obtained with the two cohorts individually largely reflect the impact of the cohort sizes on statistical power and support the use of the lists of DEGs and DMGs obtained with the full dataset in our disease connectivity analysis. In further support for this, it is noted that, of a total of 504 unique DEGs reported in previous studies[1-10], 20 had been replicated between previous studies and 117 were replicated by our study, while of a total of 322 DM CpG sites reported in 7 previous studies, 55 had been replicated between previous studies and 169 by our study (results not shown).

Use of incident breast cancer and lymphoma cases among the controls

In order to evaluate the impact of the use of incident breast cancer and lymphoma cases among our study population, we examined the influence of excluding these subjects on the statistical parameters of the significant signals (FDR<0.10 for transcriptomics, Bonferoni-corrected p<0.05 for epigenetics) obtained with the full dataset. As shown in Supplementary Fig. S2, inclusion of the incident cases results in a global and uniform increase of statistical significant ($-\log_{10}p$) and has no material effect on the effect size estimates (model regression coefficients), suggesting that inclusion of incident cases resulted in increased statistical power without causing any bias.

FIGURES

Supplementary Figure S1: Plot of $-\log_{10}$p-values, obtained with the individual cohorts as well as the pooled dataset, for the current smoker signals declared as significant (FDR<0.10 for transcriptomics, Bonferoni-corrected p<0.05 for epigenetics) in the analysis using the pooled dataset

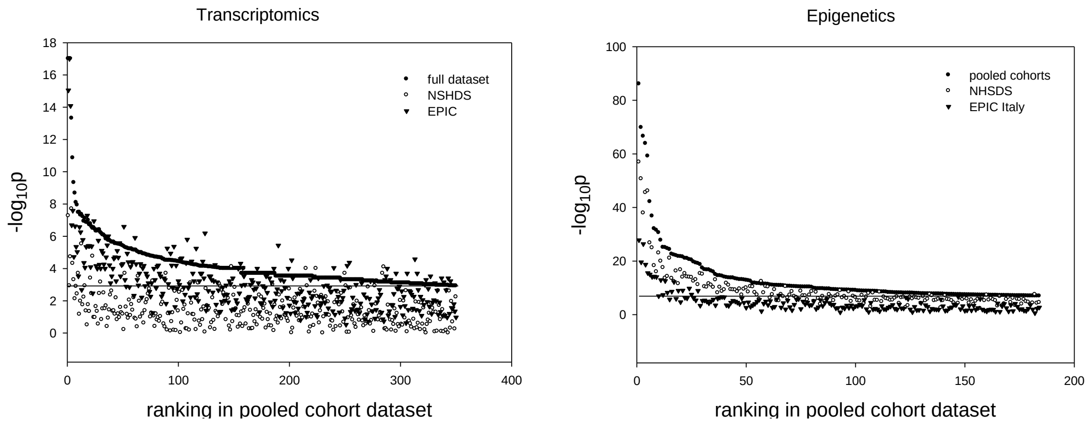

Supplementary Figure S2: Effects of inclusion of incident breast cancer and lymphoma cases on statistical parameters of epigenetic signals. Top series: transcriptomics; bottom series: epigenetics; the dashed lines represent the case of absence of any effect (slope=1)

Transcriptomics

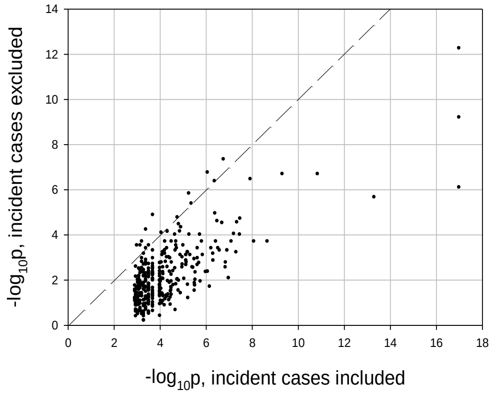

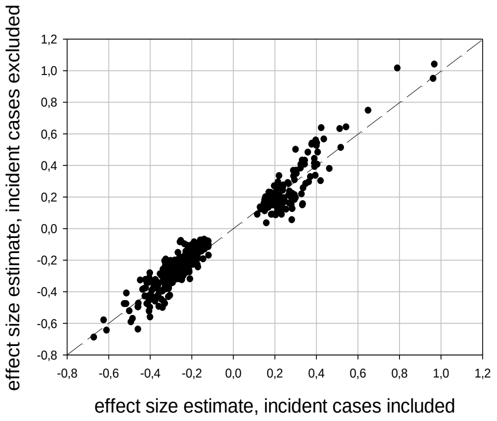

Epigenetics

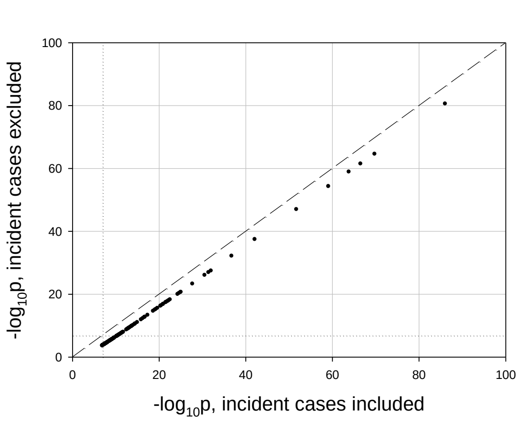

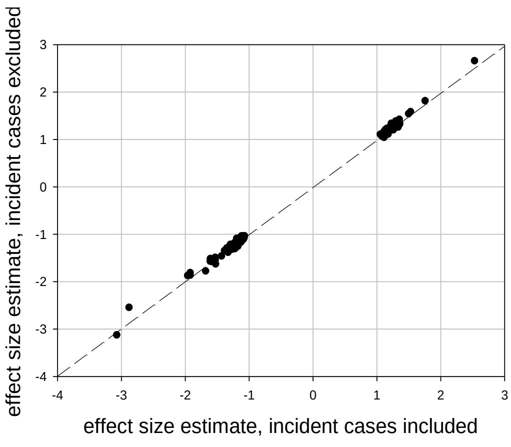

Supplementary Figure S3: Interactions between DEGs, DMGs and miRNAs related to disease categories "cancer" and "cardiovascular disease"; for the meaning of the various shapes see the legend to Fig. 3.

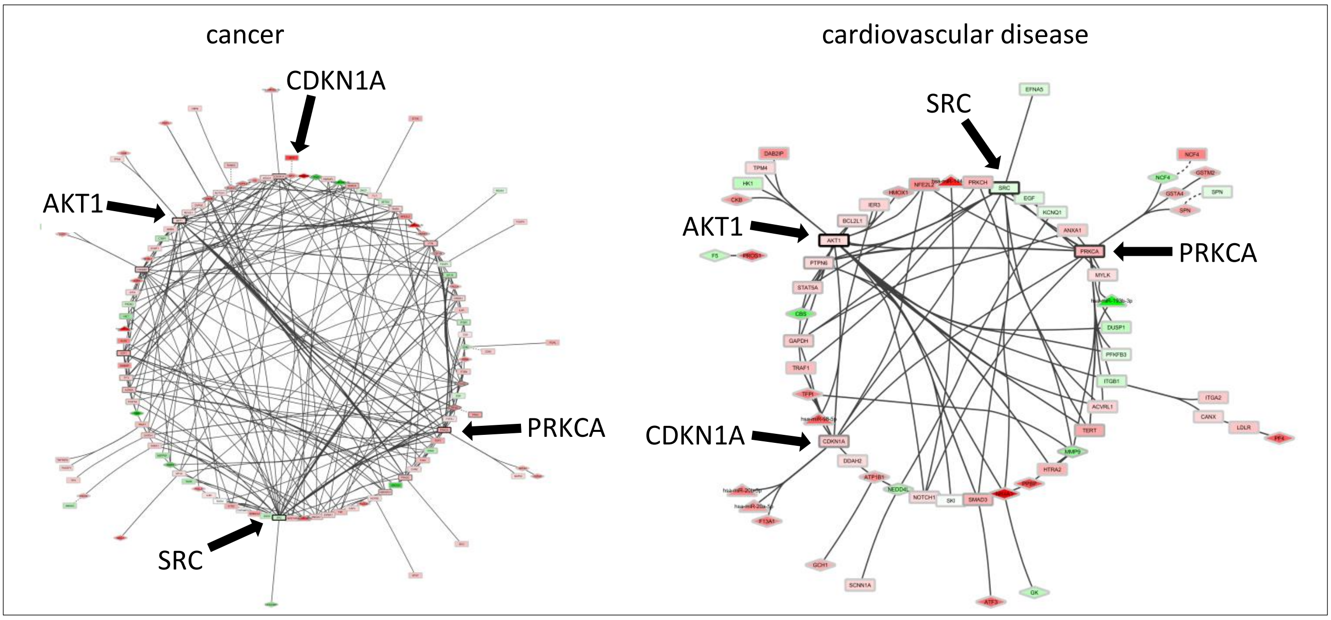

Supplementary Figure 4: Clustering of predicted disease-DEG/DMG associations

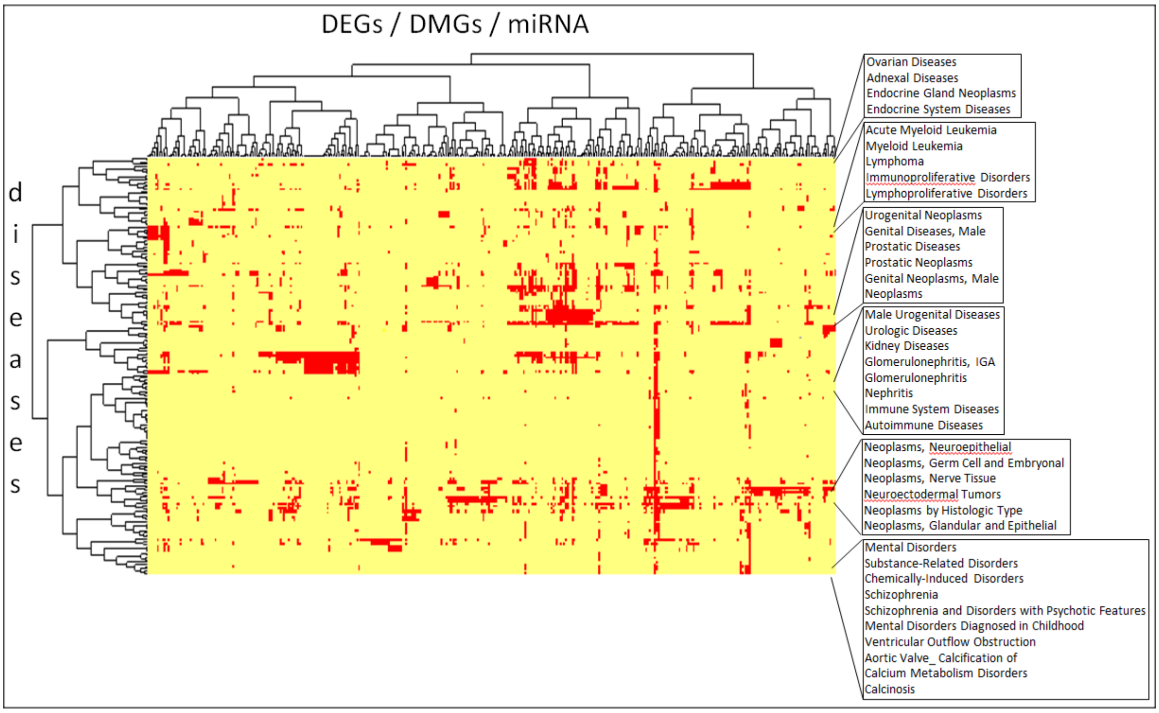

TABLES

Supplementary Table S1: Probes and associated genes differentially expressed in current smokers (FDR<0.10)

* replicated between cohorts

probe no.	gene	gene name	raw p value	FDR	foldchange expression
A_24_P187766*	LRRN3	leucine rich repeat neuronal 3	<1E-17	<1E-17	1.95
A_23_P64611	P2RY6	pyrimidinergic receptor P2Y, G-protein coupled, 6	<1E-17	<1E-17	1.38
A_23_P31376*	LRRN3	leucine rich repeat neuronal 3	<1E-17	<1E-17	1.96
A_23_P428129*	CDKN1C	cyclin-dependent kinase inhibitor 1C (p57, Kip2)	4.85E-14	3.60E-10	0.65
A_23_P115785*	FANK1	fibronectin type III and ankyrin repeat domains 1	1.39E-11	8.23E-08	1.43
A_24_P904484	LOC283174	hypothetical LOC283174	4.74E-10	2.34E-06	1.32
A_23_P150789	PRSS23	protease, serine, 23	2.13E-09	9.03E-06	0.70
A_24_P937405	PRSS23	protease, serine, 23	8.17E-09	3.03E-05	0.70
A_32_P63013	LOC283174	hypothetical LOC283174	1.16E-08	3.81E-05	1.32
A_23_P61042			3.26E-08	9.09E-05	1.57
A_23_P138492*	NEURL	neuralized homolog (Drosophila)	3.37E-08	9.09E-05	0.82
A_23_P26386	TPPP3	tubulin polymerization-promoting protein family member 3	4.46E-08	1.00E-04	0.75
A_23_P211680	MLC1	megalencephalic leukoencephalopathy with subcortical cysts 1	4.80E-08	1.00E-04	0.77
A_23_P87421			6.09E-08	1.00E-04	0.71
A_23_P253375	CUX1	cut-like homeobox 1	7.75E-08	2.00E-04	0.84
A_24_P511686	LOC100506870	hypothetical LOC100506870	1.03E-07	2.00E-04	1.23
A_23_P12514	RHOC	ras homolog gene family, member C	1.19E-07	2.00E-04	0.83
A_23_P53081	OSBPL5	oxysterol binding protein-like 5	1.37E-07	2.00E-04	0.79
A_23_P93442	SASH1	SAM and SH3 domain containing 1	1.41E-07	2.00E-04	1.32
A_23_P371276	C18orf23	chromosome 18 open reading frame 23	1.69E-07	3.00E-04	0.76
A_23_P358709	AHRR	aryl-hydrocarbon receptor repressor	1.96E-07	3.00E-04	1.73
A_23_P40847	CHST2	carbohydrate (N-acetylglucosamine-6-O) sulfotransferase 2	2.55E-07	3.00E-04	0.80
A_23_P325562	SLC1A7	solute carrier family 1 (glutamate transporter), member 7	2.94E-07	4.00E-04	0.76
A_23_P160720	BATF3	basic leucine zipper transcription factor, ATF-like 3	3.23E-07	4.00E-04	0.81
A_23_P351148	SH2D1B	SH2 domain containing 1B	3.68E-07	4.00E-04	0.75
A_24_P300379	PI16	peptidase inhibitor 16	3.99E-07	5.00E-04	1.22
A_24_P245838	MGAT3	mannosyl (beta-1,4-)-glycoprotein beta-1,4-N-acetylglucosaminyltransferase	4.18E-07	5.00E-04	1.34
A_23_P119042	NKG7	natural killer cell group 7 sequence	4.82E-07	5.00E-04	0.81
A_23_P379736	B3GNT9	UDP-GlcNAc:betaGal beta-1,3-N-acetylglucosaminyltransferase 9	4.84E-07	5.00E-04	1.15
A_23_P74088	MMP23B	matrix metallopeptidase 23B	5.85E-07	6.00E-04	0.82
A_24_P265523	CR1	complement component (3b/4b) receptor 1 (Knops blood group)	6.79E-07	6.00E-04	1.26
A_23_P107744	S1PR5	sphingosine-1-phosphate receptor 5	8.32E-07	8.00E-04	0.75
A_32_P930685	ZNF876P	zinc finger protein 876, pseudogene	8.38E-07	8.00E-04	0.80
A_23_P433760	SPN	sialophorin	1.02E-06	9.00E-04	0.88
A_24_P465799			1.37E-06	1.20E-03	1.43
A_23_P373100	MGC24103	hypothetical MGC24103	1.50E-06	1.20E-03	0.66
A_23_P254193	TTC38	tetratricopeptide repeat domain 38	1.71E-06	1.40E-03	0.80
A_23_P209700	NMUR1	neuromedin U receptor 1	1.82E-06	1.40E-03	0.79
A_23_P254654	CLIC3	chloride intracellular channel 3	2.00E-06	1.50E-03	0.80
A_32_P324533	SH2D1B	SH2 domain containing 1B	2.30E-06	1.70E-03	0.76
A_23_P152559	BZRAP1	benzodiazapine receptor (peripheral) associated protein 1	2.31E-06	1.70E-03	0.83
A_23_P206284	GPR56	G protein-coupled receptor 56	2.34E-06	1.70E-03	0.81
A_32_P171061	ASCL2	achaete-scute complex homolog 2 (Drosophila)	2.84E-06	1.90E-03	0.82
A_23_P3083			2.89E-06	1.90E-03	0.75
A_23_P206280	GPR56	G protein-coupled receptor 56	3.01E-06	2.00E-03	0.76
A_32_P190461			3.07E-06	2.00E-03	1.26
A_23_P43157	MYBL1	v-myb myeloblastosis viral oncogene homolog (avian)-like 1	3.12E-06	2.00E-03	0.81
A_32_P158966	KLRF1	killer cell lectin-like receptor subfamily F, member 1	3.18E-06	2.00E-03	0.78
A_23_P41528	FGFBP2	fibroblast growth factor binding protein 2	3.57E-06	2.20E-03	0.74
A_24_P226069	FGFBP2	fibroblast growth factor binding protein 2	3.89E-06	2.30E-03	0.75
A_23_P146554	PTGDS	prostaglandin D2 synthase 21kDa (brain)	4.26E-06	2.50E-03	0.81
A_24_P921823*	TCF7L2	transcription factor 7-like 2 (T-cell specific, HMG-box)	4.80E-06	2.70E-03	0.83
A_23_P102113	WNT10A	wingless-type MMTV integration site family, member 10A	5.24E-06	2.90E-03	1.16
A_23_P94186	LYPD2	LY6/PLAUR domain containing 2	5.44E-06	3.00E-03	0.79

A_23_P133445	GZMA	granzyme A (granzyme 1, cytotoxic T-lymphocyte-associated serine esterase 3)	5.95E-06	3.20E-03	0.80
A_32_P133916	BNC2	basonuclin 2	6.06E-06	3.20E-03	0.73
A_24_P108311	NEDD4L	neural precursor cell expressed, developmentally down-regulated 4-like	6.08E-06	3.20E-03	1.18
A_24_P169873			6.27E-06	3.20E-03	1.46
A_23_P108042	NCR1	natural cytotoxicity triggering receptor 1	6.99E-06	3.50E-03	0.79
A_23_P108404	AGAP1	ArfGAP with GTPase domain, ankyrin repeat and PH domain 1	7.27E-06	3.50E-03	0.81
A_23_P136026			7.31E-06	3.50E-03	1.33
A_23_P122662	GFOD1	glucose-fructose oxidoreductase domain containing 1	7.31E-06	3.50E-03	0.84
A_24_P79403	PF4	platelet factor 4	8.82E-06	4.20E-03	0.76
A_32_P429876	PPM1L	protein phosphatase, Mg2+/Mn2+ dependent, 1L	9.57E-06	4.40E-03	0.80
A_24_P88850	MRAS	muscle RAS oncogene homolog	1.04E-05	4.70E-03	0.81
A_23_P203351	MS4A7	membrane-spanning 4-domains, subfamily A, member 7	1.05E-05	4.70E-03	0.83
A_23_P128230	NR4A1	nuclear receptor subfamily 4, group A, member 1	1.07E-05	4.70E-03	0.63
A_32_P15035	GFOD1	glucose-fructose oxidoreductase domain containing 1	1.22E-05	5.30E-03	0.84
A_23_P141555	TBX21	T-box 21	1.25E-05	5.40E-03	0.80
A_23_P372496	DNAH12	dynein, axonemal, heavy chain 12	1.29E-05	5.50E-03	0.71
A_23_P155556	CLDND1	claudin domain containing 1	1.35E-05	5.60E-03	1.15
A_32_P164593	ZMAT4	zinc finger, matrin-type 4	1.50E-05	6.10E-03	0.70
A_23_P50946	RAMP1	receptor (G protein-coupled) activity modifying protein 1	1.51E-05	6.10E-03	0.81
A_23_P16976	ANXA4	annexin A4	1.56E-05	6.30E-03	0.87
A_23_P121596	PPBP	pro-platelet basic protein (chemokine (C-X-C motif) ligand 7)	1.71E-05	6.70E-03	0.74
A_23_P61987	TMEM121	transmembrane protein 121	1.72E-05	6.70E-03	1.17
A_23_P120883	HMOX1	heme oxygenase (decycling) 1	1.77E-05	6.80E-03	0.84
A_23_P70719	LAMA2	laminin, alpha 2	1.85E-05	7.00E-03	0.82
A_23_P254507	HOPX	HOP homeobox	1.96E-05	7.20E-03	0.85
A_24_P154080	ECE1	endothelin converting enzyme 1	1.97E-05	7.20E-03	1.18
A_24_P519504			1.97E-05	7.20E-03	1.36
A_24_P353619	ALPL	alkaline phosphatase, liver/bone/kidney	2.11E-05	7.50E-03	1.26
A_23_P121533	SPON2	spondin 2, extracellular matrix protein	2.11E-05	7.50E-03	0.77
A_23_P110941	GSTA4	glutathione S-transferase alpha 4	2.18E-05	7.60E-03	0.87
A_23_P406424	RHOC	ras homolog gene family, member C	2.19E-05	7.60E-03	0.91
A_24_P156388	TTC38	tetratricopeptide repeat domain 38	2.60E-05	9.00E-03	0.82
A_24_P913146	HOPX	HOP homeobox	2.65E-05	9.10E-03	0.84
A_23_P136753			3.09E-05	1.00E-02	1.20
A_23_P381714	CA13	carbonic anhydrase XIII	3.10E-05	1.00E-02	0.84
A_23_P346900	CACNA2D2	calcium channel, voltage-dependent, alpha 2/delta subunit 2	3.12E-05	1.00E-02	0.84
A_23_P26994	GNGT2	guanine nucleotide binding protein (G protein), gamma transducing activity polypeptide 2	3.13E-05	1.00E-02	0.86
A_23_P354341	CD160	CD160 molecule	3.14E-05	1.00E-02	0.78
A_32_P87191	FLJ21408	hypothetical LOC400512	3.14E-05	1.00E-02	1.29
A_23_P56578	VIT	vitrin	3.23E-05	1.02E-02	0.76
A_23_P117602	GZMB	granzyme B (granzyme 2, cytotoxic T-lymphocyte-associated serine esterase 1)	3.26E-05	1.02E-02	0.80
A_23_P21485	PID1	phosphotyrosine interaction domain containing 1	3.36E-05	1.04E-02	1.18
A_23_P138117	CAMTA1	calmodulin binding transcription activator 1	3.42E-05	1.05E-02	0.87
A_23_P97046	ALPL	alkaline phosphatase, liver/bone/kidney	3.45E-05	1.05E-02	1.22
A_32_P133564			3.61E-05	1.08E-02	1.25
A_23_P1833	B3GAT1	beta-1,3-glucuronyltransferase 1 (glucuronosyltransferase P)	3.73E-05	1.10E-02	0.77
A_24_P175435	SLC2A8	solute carrier family 2 (facilitated glucose transporter), member 8	3.76E-05	1.10E-02	0.88
A_23_P397671	CR1	complement component (3b/4b) receptor 1 (Knops blood group)	3.97E-05	1.15E-02	1.23
A_32_P12232	BNC2	basonuclin 2	4.04E-05	1.16E-02	0.72
A_24_P173754	C1orf21	chromosome 1 open reading frame 21	4.36E-05	1.24E-02	0.82
A_24_P240166	PHLDB2	pleckstrin homology-like domain, family B, member 2	4.50E-05	1.27E-02	0.84
A_24_P395415			4.67E-05	1.30E-02	1.30
A_23_P253221	ARHGEF4	Rho guanine nucleotide exchange factor (GEF) 4	4.69E-05	1.30E-02	1.16
A_23_P4494	DSC2	desmocollin 2	4.78E-05	1.31E-02	1.34
A_24_P362805	GK5	glycerol kinase 5 (putative)	4.93E-05	1.34E-02	0.86
A_23_P406385	FBXL16	F-box and leucine-rich repeat protein 16	4.99E-05	1.35E-02	1.17
A_32_P69368	ID2	inhibitor of DNA binding 2, dominant negative helix-loop-helix protein	5.27E-05	1.41E-02	0.86
A_32_P26721			5.59E-05	1.48E-02	0.88
A_32_P166693	HEG1	HEG homolog 1 (zebrafish)	5.74E-05	1.51E-02	0.87
A_23_P112482	AQP3	aquaporin 3 (Gill blood group)	5.96E-05	1.55E-02	1.16
A_23_P1331	COL13A1	collagen, type XIII, alpha 1	6.03E-05	1.55E-02	0.76
A_23_P113748	ZNF385D	zinc finger protein 385D	6.05E-05	1.55E-02	0.76
A_23_P57347	PCNT	pericentrin	6.22E-05	1.58E-02	0.90

A_23_P213102	PALLD	palladin, cytoskeletal associated protein	6.55E-05	1.65E-02	0.83
A_23_P200780	TGFBR3	transforming growth factor, beta receptor III	7.03E-05	1.75E-02	0.83
A_24_P272993	MEG3	maternally expressed 3 (non-protein coding)	7.11E-05	1.75E-02	0.73
A_24_P203000	IL2RB	interleukin 2 receptor, beta	7.16E-05	1.75E-02	0.84
A_23_P202881	FEZ1	fasciculation and elongation protein zeta 1 (zygin I)	7.24E-05	1.75E-02	0.82
A_24_P658584	SASH1	SAM and SH3 domain containing 1	7.28E-05	1.75E-02	1.31
A_24_P230563	IL2RA	interleukin 2 receptor, alpha	7.30E-05	1.75E-02	1.18
A_23_P418015	MAPRE2	microtubule-associated protein, RP/EB family, member 2	7.50E-05	1.77E-02	0.88
A_23_P69171	SUCNR1	succinate receptor 1	7.50E-05	1.77E-02	0.77
A_24_P417706	MXD3	MAX dimerization protein 3	7.81E-05	1.81E-02	1.20
A_23_P23834	LGR6	leucine-rich repeat-containing G protein-coupled receptor 6	7.87E-05	1.81E-02	0.78
A_23_P136347	EPS8	epidermal growth factor receptor pathway substrate 8	7.87E-05	1.81E-02	0.81
A_23_P93524	SAMD3	sterile alpha motif domain containing 3	8.11E-05	1.85E-02	0.83
A_23_P25674	CKB	creatine kinase, brain	8.37E-05	1.89E-02	0.84
A_23_P327380	TP63	tumor protein p63	8.41E-05	1.89E-02	1.27
A_23_P211039	ADAMTS1	ADAM metallopeptidase with thrombospondin type 1 motif, 1	8.59E-05	1.92E-02	0.79
A_24_P417460	SFMBT2	Scm-like with four mbt domains 2	8.75E-05	1.94E-02	0.81
A_23_P113161	C1orf21	chromosome 1 open reading frame 21	9.05E-05	1.99E-02	0.80
A_32_P129310			9.21E-05	2.01E-02	1.15
A_23_P32233	KLF4	Kruppel-like factor 4 (gut)	9.41E-05	2.04E-02	0.85
A_23_P33768	ZFYVE9	zinc finger, FYVE domain containing 9	9.98E-05	2.13E-02	1.16
A_24_P13041	RTKN2	rhotekin 2	9.99E-05	2.13E-02	1.24
A_23_P143143	ID2	inhibitor of DNA binding 2, dominant negative helix-loop-helix protein	1.00E-04	2.13E-02	0.85
A_24_P113131	BZRAP1	benzodiazapine receptor (peripheral) associated protein 1	1.00E-04	2.17E-02	0.84
A_32_P34404			1.00E-04	2.20E-02	0.86
A_23_P203920	SSPN	sarcospan (Kras oncogene-associated gene)	1.00E-04	2.20E-02	1.18
A_23_P366366	SCRN1	secernin 1	1.00E-04	2.29E-02	0.87
A_23_P146943	ATP1B1	ATPase, Na+/K+ transporting, beta 1 polypeptide	1.00E-04	2.30E-02	0.84
A_23_P353436			1.00E-04	2.32E-02	0.87
A_24_P583225	NCRNA00265	non-protein coding RNA 265	1.00E-04	2.42E-02	1.18
A_23_P44505	KLF11	Kruppel-like factor 11	1.00E-04	2.48E-02	0.87
A_23_P53193	SYTL2	synaptotagmin-like 2	1.00E-04	2.49E-02	0.83
A_24_P343233	HLA-DRB1	major histocompatibility complex, class II, DR beta 1	1.00E-04	2.49E-02	0.89
A_23_P138706	ADRA2A	adrenergic, alpha-2A-, receptor	1.00E-04	2.69E-02	0.81
A_23_P107750	S1PR5	sphingosine-1-phosphate receptor 5	1.00E-04	2.77E-02	0.80
A_23_P151120	ACRBP	acrosin binding protein	1.00E-04	2.77E-02	0.81
A_23_P127948	ADM	adrenomedullin	1.00E-04	2.78E-02	1.30
A_23_P22096	PTK2	PTK2 protein tyrosine kinase 2	1.00E-04	2.81E-02	0.84
A_24_P399220	HOXB3	homeobox B3	1.00E-04	2.82E-02	0.82
A_23_P16006	ZNF600	zinc finger protein 600	1.00E-04	2.82E-02	0.87
A_23_P257365	GFI1	growth factor independent 1 transcription repressor	2.00E-04	2.82E-02	0.82
A_24_P173566			2.00E-04	2.82E-02	1.24
A_24_P179816	SLC27A3	solute carrier family 27 (fatty acid transporter), member 3	2.00E-04	2.83E-02	0.90
A_23_P72989	CCR4	chemokine (C-C motif) receptor 4	2.00E-04	2.83E-02	1.18
A_23_P112452	GGTA1	glycoprotein, alpha-galactosyltransferase 1 pseudogene	2.00E-04	2.95E-02	0.78
A_32_P50406	FLJ45340	hypothetical LOC402483	2.00E-04	3.03E-02	1.17
A_24_P184803	COCH	coagulation factor C homolog, cochlin (Limulus polyphemus)	2.00E-04	3.03E-02	1.30
A_23_P98565	MS4A14	membrane-spanning 4-domains, subfamily A, member 14	2.00E-04	3.11E-02	0.80
A_24_P24890			2.00E-04	3.27E-02	1.31
A_23_P72252			2.00E-04	3.29E-02	1.23
A_23_P200685	MOSC2	MOCO sulphurase C-terminal domain containing 2	2.00E-04	3.31E-02	1.17
A_24_P234554	ZNF385D	zinc finger protein 385D	2.00E-04	3.34E-02	0.73
A_23_P43684	BNC2	basonuclin 2	2.00E-04	3.43E-02	0.82
A_32_P196669			2.00E-04	3.43E-02	1.19
A_23_P51231	RUNX3	runt-related transcription factor 3	2.00E-04	3.45E-02	0.87
A_23_P8913	CA2	carbonic anhydrase II	2.00E-04	3.47E-02	0.81
A_23_P86682	MYOF	myoferlin	2.00E-04	3.47E-02	0.83
A_24_P589028			2.00E-04	3.55E-02	0.73
A_23_P121676	CXXC4	CXXC finger protein 4	2.00E-04	3.55E-02	0.76
A_23_P206290	GPR114	G protein-coupled receptor 114	2.00E-04	3.55E-02	0.86
A_23_P43979			2.00E-04	3.55E-02	1.23
A_23_P66881	RGS9	regulator of G-protein signaling 9	2.00E-04	3.62E-02	0.81
A_23_P39251	PLIN5	perilipin 5	2.00E-04	3.62E-02	1.23
A_23_P99275	KLRB1	killer cell lectin-like receptor subfamily B, member 1	2.00E-04	3.67E-02	0.85
A_23_P109508	NCF4	neutrophil cytosolic factor 4, 40kDa	2.00E-04	3.72E-02	1.21
A_23_P385105	PLCD4	phospholipase C, delta 4	2.00E-04	3.88E-02	0.85

A_24_P267814	LOC100287891	hypothetical LOC100287891	2.00E-04	3.88E-02	1.26
A_23_P201376	SSX2IP	synovial sarcoma, X breakpoint 2 interacting protein	2.00E-04	3.90E-02	0.81
A_24_P743802	ZNF618	zinc finger protein 618	2.00E-04	3.90E-02	0.84
A_23_P39931	DYSF	dysferlin, limb girdle muscular dystrophy 2B (autosomal recessive)	2.00E-04	3.90E-02	1.17
A_24_P141481	CD59	CD59 molecule, complement regulatory protein	3.00E-04	3.96E-02	1.10
A_23_P109171	BFSP1	beaded filament structural protein 1, filensin	3.00E-04	3.97E-02	0.82
A_23_P336612	C15orf26	chromosome 15 open reading frame 26	3.00E-04	3.98E-02	0.78
A_23_P347632	MTSS1	metastasis suppressor 1	3.00E-04	3.98E-02	0.88
A_23_P259506	C5orf32	chromosome 5 open reading frame 32	3.00E-04	3.98E-02	1.18
A_23_P213620	PPP2R2B	protein phosphatase 2, regulatory subunit B, beta	3.00E-04	4.02E-02	0.86
A_23_P348257	NUAK1	NUAK family, SNF1-like kinase, 1	3.00E-04	4.03E-02	0.80
A_23_P127128	DNAJC1	DnaJ (Hsp40) homolog, subfamily C, member 1	3.00E-04	4.03E-02	0.90
A_23_P99614	BTBD6	BTB (POZ) domain containing 6	3.00E-04	4.03E-02	0.92
A_23_P48212	CLEC1B	C-type lectin domain family 1, member B	3.00E-04	4.07E-02	0.79
A_24_P139604	PYHIN1	pyrin and HIN domain family, member 1	3.00E-04	4.07E-02	0.83
A_32_P75141			3.00E-04	4.07E-02	0.85
A_24_P339560	SIGLEC11	sialic acid binding Ig-like lectin 11	3.00E-04	4.07E-02	1.26
A_23_P328740	NEURL3	neuralized homolog 3 (Drosophila) pseudogene	3.00E-04	4.09E-02	0.78
A_23_P89249	ERBB2	v-erb-b2 erythroblastic leukemia viral oncogene homolog 2, neuro/glioblastoma derived oncogene homolog (avian)	3.00E-04	4.09E-02	0.89
A_23_P374902	CLDND2	claudin domain containing 2	3.00E-04	4.19E-02	0.86
A_24_P383523	SAMD4A	sterile alpha motif domain containing 4A	3.00E-04	4.20E-02	0.89
A_23_P209527	VIL1	villin 1	3.00E-04	4.28E-02	0.77
A_24_P466590			3.00E-04	4.28E-02	0.88
A_24_P104980	IGLL5	immunoglobulin lambda-like polypeptide 5	3.00E-04	4.28E-02	1.17
A_24_P119141	PROS1	protein S (alpha)	3.00E-04	4.34E-02	0.75
A_23_P397937	SAMD3	sterile alpha motif domain containing 3	3.00E-04	4.41E-02	0.85
A_24_P166443	HLA-DPB1	major histocompatibility complex, class II, DP beta 1	3.00E-04	4.50E-02	0.88
A_23_P13232			3.00E-04	4.53E-02	0.80
A_23_P157569	ADHFE1	alcohol dehydrogenase, iron containing, 1	3.00E-04	4.53E-02	0.87
A_23_P85453	CD244	CD244 molecule, natural killer cell receptor 2B4	3.00E-04	4.53E-02	0.88
A_24_P274219	EPHA4	EPH receptor A4	3.00E-04	4.53E-02	1.14
A_24_P940166	PAPSS2	3'-phosphoadenosine 5'-phosphosulfate synthase 2	3.00E-04	4.55E-02	0.83
A_23_P397208	GSTM2	glutathione S-transferase mu 2 (muscle)	3.00E-04	4.55E-02	0.85
A_32_P25639	BET3L	BET3 like (S. cerevisiae)	3.00E-04	4.57E-02	0.79
A_23_P342275	ADAMTS1	ADAM metallopeptidase with thrombospondin type 1 motif, 1	3.00E-04	4.64E-02	0.80
A_23_P205200	DHRS12	dehydrogenase/reductase (SDR family) member 12	3.00E-04	4.64E-02	1.14
A_24_P396167	CTSW	cathepsin W	3.00E-04	4.67E-02	0.84
A_23_P216094	ASPH	aspartate beta-hydroxylase	3.00E-04	4.68E-02	1.14
A_23_P17633	IFNAR1	interferon (alpha, beta and omega) receptor 1	4.00E-04	4.70E-02	1.12
A_23_P434118	CEACAM1	carcinoembryonic antigen-related cell adhesion molecule 1 (biliary glycoprotein)	4.00E-04	4.74E-02	1.16
A_23_P154526	GRB14	growth factor receptor-bound protein 14	4.00E-04	4.78E-02	0.79
A_23_P214244	ENPP5	ectonucleotide pyrophosphatase/phosphodiesterase 5 (putative)	4.00E-04	4.78E-02	0.80
A_23_P163697	SYT17	synaptotagmin XVII	4.00E-04	4.78E-02	0.86
A_23_P101093	COPZ2	coatomer protein complex, subunit zeta 2	4.00E-04	4.78E-02	0.86
A_23_P72050	PTK2	PTK2 protein tyrosine kinase 2	4.00E-04	4.78E-02	0.86
A_32_P163147	VSIG1	V-set and immunoglobulin domain containing 1	4.00E-04	4.78E-02	1.10
A_32_P150012	CHD7	chromodomain helicase DNA binding protein 7	4.00E-04	4.97E-02	1.12
A_24_P184799	COCH	coagulation factor C homolog, cochlin (Limulus polyphemus)	4.00E-04	4.98E-02	1.32
A_23_P205370	ASB2	ankyrin repeat and SOCS box-containing 2	4.00E-04	5.04E-02	1.13
A_23_P31006	HLA-DRB5	major histocompatibility complex, class II, DR beta 5	4.00E-04	5.11E-02	0.86
A_24_P414169	TFDP2	transcription factor Dp-2 (E2F dimerization partner 2)	4.00E-04	5.17E-02	0.89
A_24_P129632	DLG5	discs, large homolog 5 (Drosophila)	4.00E-04	5.22E-02	0.80
A_23_P128728	ARG2	arginase, type II	4.00E-04	5.22E-02	0.81
A_23_P19987	IGF2BP3	insulin-like growth factor 2 mRNA binding protein 3	4.00E-04	5.22E-02	0.82
A_23_P104493	PAPSS2	3'-phosphoadenosine 5'-phosphosulfate synthase 2	4.00E-04	5.22E-02	0.84
A_23_P400378	GPBAR1	G protein-coupled bile acid receptor 1	4.00E-04	5.22E-02	0.89
A_23_P115417	RGL1	ral guanine nucleotide dissociation stimulator-like 1	4.00E-04	5.22E-02	1.11
A_32_P139163			4.00E-04	5.22E-02	1.15
A_23_P156826	C6orf105	chromosome 6 open reading frame 105	4.00E-04	5.22E-02	1.21
A_23_P256821	CR1	complement component (3b/4b) receptor 1 (Knops blood group)	4.00E-04	5.22E-02	1.22
A_23_P421323			4.00E-04	5.22E-02	1.28
A_23_P24193	XPNPEP1	X-prolyl aminopeptidase (aminopeptidase P) 1, soluble	4.00E-04	5.25E-02	0.88
A_23_P138541	AKR1C3	aldo-keto reductase family 1, member C3 (3-alpha hydroxysteroid dehydrogenase, type II)	4.00E-04	5.33E-02	0.81
A_23_P158925	GPR125	G protein-coupled receptor 125	5.00E-04	5.60E-02	0.80
A_23_P380614	ATP9A	ATPase, class II, type 9A	5.00E-04	5.60E-02	0.83

A_23_P75325	SGPL1	sphingosine-1-phosphate lyase 1	5.00E-04	5.60E-02	0.88
A_23_P435390			5.00E-04	5.60E-02	1.27
A_23_P412214	RAP1GAP2	RAP1 GTPase activating protein 2	5.00E-04	5.61E-02	0.78
A_23_P56213	GRAMD1A	GRAM domain containing 1A	5.00E-04	5.61E-02	1.11
A_24_P342178			5.00E-04	5.66E-02	1.12
A_23_P48175	TMEM106C	transmembrane protein 106C	5.00E-04	5.72E-02	0.88
A_23_P1782	CD82	CD82 molecule	5.00E-04	5.72E-02	1.11
A_23_P413456	CIRBP	cold inducible RNA binding protein	5.00E-04	5.72E-02	1.18
A_23_P123413	TOX	thymocyte selection-associated high mobility group box	5.00E-04	5.73E-02	0.84
A_32_P88120	YPEL1	yippee-like 1 (Drosophila)	5.00E-04	5.75E-02	0.90
A_24_P472081			5.00E-04	5.75E-02	1.18
A_32_P156786			5.00E-04	5.75E-02	1.22
A_23_P155939	ZNF595	zinc finger protein 595	5.00E-04	6.03E-02	0.84
A_32_P89269			5.00E-04	6.05E-02	0.81
A_23_P418373	BCL2L2	BCL2-like 2	5.00E-04	6.09E-02	0.89
A_23_P170534	FUT7	fucosyltransferase 7 (alpha (1,3) fucosyltransferase)	5.00E-04	6.12E-02	1.15
A_23_P397391	FFAR2	free fatty acid receptor 2	5.00E-04	6.12E-02	1.22
A_23_P167168	IGJ	immunoglobulin J polypeptide, linker protein for immunoglobulin alpha and mu polypeptides	5.00E-04	6.12E-02	1.24
A_24_P83102	IGLL1	immunoglobulin lambda-like polypeptide 1	6.00E-04	6.13E-02	1.26
A_24_P565503			6.00E-04	6.19E-02	1.14
A_32_P43664			6.00E-04	6.19E-02	1.29
A_24_P409971	NEXN	nexilin (F actin binding protein)	6.00E-04	6.26E-02	0.82
A_24_P88763	LOXL3	lysyl oxidase-like 3	6.00E-04	6.28E-02	0.89
A_23_P52451	HKDC1	hexokinase domain containing 1	6.00E-04	6.49E-02	1.17
A_24_P940288	PGS1	phosphatidylglycerophosphate synthase 1	6.00E-04	6.49E-02	1.18
A_23_P1473	PRF1	perforin 1 (pore forming protein)	6.00E-04	6.52E-02	0.82
A_24_P410453	SYNE1	spectrin repeat containing, nuclear envelope 1	6.00E-04	6.52E-02	0.84
A_24_P246573	KAZ	kazrin	6.00E-04	6.68E-02	1.31
A_23_P34045	EDA	ectodysplasin A	6.00E-04	6.92E-02	1.13
A_23_P58796	RGMB	RGM domain family, member B	6.00E-04	6.92E-02	1.23
A_23_P168828	KLF10	Kruppel-like factor 10	7.00E-04	6.92E-02	0.84
A_32_P140139	F13A1	coagulation factor XIII, A1 polypeptide	7.00E-04	6.96E-02	0.82
A_24_P178602	ZNF600	zinc finger protein 600	7.00E-04	6.96E-02	0.85
A_23_P2661	RAP1B	RAP1B, member of RAS oncogene family	7.00E-04	6.96E-02	0.90
A_24_P298877	C1orf174	chromosome 1 open reading frame 174	7.00E-04	6.96E-02	0.91
A_24_P302406			7.00E-04	6.97E-02	0.90
A_24_P76644			7.00E-04	7.11E-02	0.90
A_23_P38830	ZNF552	zinc finger protein 552	7.00E-04	7.12E-02	1.14
A_23_P166306	CBS	cystathionine-beta-synthase	7.00E-04	7.17E-02	1.33
A_23_P43107	TM7SF4	transmembrane 7 superfamily member 4	7.00E-04	7.18E-02	0.80
A_23_P17095	TFPI	tissue factor pathway inhibitor (lipoprotein-associated coagulation inhibitor)	7.00E-04	7.54E-02	0.82
A_23_P132226	TPST2	tyrosylprotein sulfotransferase 2	7.00E-04	7.60E-02	0.90
A_23_P218225	QPRT	quinolinate phosphoribosyltransferase	7.00E-04	7.61E-02	0.92
A_23_P119794			8.00E-04	7.65E-02	0.86
A_23_P211550	RBX1	ring-box 1, E3 ubiquitin protein ligase	8.00E-04	7.65E-02	0.92
A_23_P386241	FAM110A	family with sequence similarity 110, member A	8.00E-04	7.66E-02	0.91
A_23_P88351	ATL1	atlastin GTPase 1	8.00E-04	7.80E-02	0.88
A_23_P159237	GPR20	G protein-coupled receptor 20	8.00E-04	7.86E-02	0.87
A_24_P755069			8.00E-04	7.90E-02	0.85
A_23_P13031	CTSW	cathepsin W	8.00E-04	8.06E-02	0.86
A_23_P45831	CHD1L	chromodomain helicase DNA binding protein 1-like	8.00E-04	8.06E-02	0.92
A_23_P112957			8.00E-04	8.06E-02	1.13
A_23_P100704	MAPK7	mitogen-activated protein kinase 7	8.00E-04	8.08E-02	0.92
A_23_P302550	RGS18	regulator of G-protein signaling 18	8.00E-04	8.09E-02	0.82
A_23_P43679	ZNF618	zinc finger protein 618	8.00E-04	8.09E-02	0.89
A_23_P3450	TUBGCP4	tubulin, gamma complex associated protein 4	8.00E-04	8.09E-02	0.92
A_23_P312932	KRTAP8-1	keratin associated protein 8-1	8.00E-04	8.09E-02	1.20
A_24_P272222	PLIN5	perilipin 5	8.00E-04	8.12E-02	1.16
A_23_P209129	LAIR2	leukocyte-associated immunoglobulin-like receptor 2	8.00E-04	8.17E-02	0.73
A_23_P139486	CDK2AP1	cyclin-dependent kinase 2 associated protein 1	8.00E-04	8.17E-02	0.89
A_23_P9485	ORM2	orosomucoid 2	9.00E-04	8.30E-02	1.22
A_23_P57413	PPM1F	protein phosphatase, Mg2+/Mn2+ dependent, 1F	9.00E-04	8.43E-02	1.13
A_23_P34915	ATF3	activating transcription factor 3	9.00E-04	8.47E-02	0.78
A_23_P428887	KLHL34	kelch-like 34 (Drosophila)	9.00E-04	8.47E-02	1.12
A_23_P116512	PRR5L	proline rich 5 like	9.00E-04	8.55E-02	0.83
A_32_P140475	KIAA1377	KIAA1377	9.00E-04	8.66E-02	0.88

A_23_P212779	PARM1	prostate androgen-regulated mucin-like protein 1	9.00E-04	8.66E-02	1.14
A_32_P172848	GK	glycerol kinase	9.00E-04	8.66E-02	1.15
A_32_P90615			9.00E-04	8.66E-02	1.18
A_32_P54289			9.00E-04	8.72E-02	1.19
A_23_P1962	RARRES3	retinoic acid receptor responder (tazarotene induced) 3	9.00E-04	8.74E-02	0.90
A_32_P187009	SERINC5	serine incorporator 5	9.00E-04	8.75E-02	1.12
A_24_P367227	MYBL1	v-myb myeloblastosis viral oncogene homolog (avian)-like 1	1.00E-03	9.03E-02	0.84
A_23_P377267			1.00E-03	9.03E-02	0.90
A_23_P66719	DHRS13	dehydrogenase/reductase (SDR family) member 13	1.00E-03	9.03E-02	1.14
A_23_P40174	MMP9	matrix metallopeptidase 9 (gelatinase B, 92kDa gelatinase, 92kDa type IV collagenase)	1.00E-03	9.03E-02	1.23
A_32_P32195			1.00E-03	9.05E-02	0.87
A_23_P217510	CD99	CD99 molecule	1.00E-03	9.05E-02	0.91
A_23_P148768	F5	coagulation factor V (proaccelerin, labile factor)	1.00E-03	9.05E-02	1.15
A_24_P355693	ACER3	alkaline ceramidase 3	1.00E-03	9.23E-02	0.86
A_23_P101407	C3	complement component 3	1.00E-03	9.24E-02	0.89
A_23_P40548	YPEL1	yippee-like 1 (Drosophila)	1.00E-03	9.24E-02	0.91
A_32_P220897			1.00E-03	9.40E-02	1.11
A_24_P124349	PDGFD	platelet derived growth factor D	1.10E-03	9.56E-02	0.80
A_32_P28158			1.10E-03	9.70E-02	1.18
A_23_P87013	TAGLN	transgelin	1.10E-03	9.71E-02	0.86
A_32_P141768	AGPAT4	1-acylglycerol-3-phosphate O-acyltransferase 4 (lysophosphatidic acid acyltransferase, delta)	1.10E-03	9.71E-02	0.88
A_23_P135123			1.10E-03	9.77E-02	0.82
A_24_P353638	SLAMF7	SLAM family member 7	1.10E-03	9.77E-02	0.87
A_24_P266734	SSH3	slingshot homolog 3 (Drosophila)	1.10E-03	9.77E-02	1.09
A_23_P209625	CYP1B1	cytochrome P450, family 1, subfamily B, polypeptide 1	1.10E-03	9.77E-02	1.19
A_23_P211910	PLOD2	procollagen-lysine, 2-oxoglutarate 5-dioxygenase 2	1.10E-03	9.79E-02	0.80
A_24_P9883	DKFZp761E198	DKFZp761E198 protein	1.10E-03	9.79E-02	1.22
A_23_P354387	MYOF	myoferlin	1.10E-03	9.80E-02	0.85
A_23_P39925	DYSF	dysferlin, limb girdle muscular dystrophy 2B (autosomal recessive)	1.10E-03	9.80E-02	1.17
A_23_P45917	CKS1B	CDC28 protein kinase regulatory subunit 1B	1.10E-03	9.85E-02	0.91
A_24_P215240	ENKUR	enkurin, TRPC channel interacting protein	1.20E-03	9.85E-02	0.80
A_23_P110791	CSF1R	colony stimulating factor 1 receptor	1.20E-03	9.85E-02	0.87
A_24_P167642	GCH1	GTP cyclohydrolase 1	1.20E-03	9.85E-02	0.88
A_23_P130836	GZMM	granzyme M (lymphocyte met-ase 1)	1.20E-03	9.85E-02	0.91
A_24_P169843			1.20E-03	9.85E-02	1.16
A_23_P43810	LTBP1	latent transforming growth factor beta binding protein 1	1.20E-03	9.99E-02	0.79

Supplementary Table S2: CpG sites and associated genes differentially methylated in current smokers

(Bonferoni-corrected p<0.05)

* replicated between cohorts

CpG site	gene	gene name	raw p value	FDR	β (methylation). smokers (%)	β (methylation). never smokers (%)	Δβ
cg05575921*	AHRR	aryl-hydrocarbon receptor repressor	8.1E-87	3.33E-81	53.72	78.06	-24.34
cg21566642*			1.52E-70	3.12E-65	29.85	44.88	-15.03
cg03636183*	F2RL3	coagulation factor II (thrombin) receptor-like 3	2.8E-67	3.83E-62	55.04	65.85	-10.81
cg01940273*			1.38E-64	1.42E-59	47.05	57.5	-10.45
cg05951221*			7.13E-60	5.86E-55	28.78	39.28	-10.51
cg06126421*			7.27E-43	4.27E-38	52.23	62.46	-10.23
cg26703534*	AHRR	aryl-hydrocarbon receptor repressor	1.64E-37	8.41E-33	57.34	62.53	-5.19
cg25648203*	AHRR	aryl-hydrocarbon receptor repressor	9.41E-33	4.3E-28	71.73	76.3	-4.57
cg15342087*			3.41E-32	1.4E-27	76.07	79.98	-3.91
cg03329539*			2.74E-31	1.02E-26	29.03	33.44	-4.40
cg19859270	GPR15	G protein-coupled receptor 15	1.78E-28	6.1E-24	70.35	74.91	-4.56
cg09935388*	GFI1	growth factor independent 1 transcription repressor	8.36E-26	2.64E-21	61.19	69.18	-7.98
cg14753356*			1E-25	2.94E-21	29.36	33.25	-3.90
cg27537125*			2.17E-25	5.94E-21	10.32	11.9	-1.58
cg14817490*	AHRR	aryl-hydrocarbon receptor repressor	4.7E-25	1.21E-20	16.83	20.93	-4.10
cg24859433*			2.75E-23	6.65E-19	74.97	78.58	-3.61
cg21611682*	LRP5	low density lipoprotein receptor-related protein 5	5.49E-23	1.25E-18	48.85	51.86	-3.01
cg22132788*	MYO1G	myosin IG	9.41E-23	2.03E-18	87.8	73.93	13.87
cg19572487	RARA	retinoic acid receptor. alpha	1.99E-22	4.1E-18	43.24	48.25	-5.01
cg06644428			2.19E-22	4.28E-18	2.74	4.31	-1.57
cg23079012*			3E-22	5.61E-18	85.99	94.63	-8.64
cg12803068*	MYO1G	myosin IG	9.56E-22	1.71E-17	74.56	62.41	12.14
cg23161492*	ANPEP	alanyl (membrane) aminopeptidase	1.15E-21	1.97E-17	18.05	22.5	-4.45
cg25949550*	CNTNAP2	contactin associated protein-like 2	2.78E-21	4.58E-17	7.12	8.61	-1.49
cg23576855*	AHRR	aryl-hydrocarbon receptor repressor	4.28E-21	6.77E-17	41.15	57.24	-16.09
cg21322436	CNTNAP2	contactin associated protein-like 2	2.23E-20	3.39E-16	19	21.84	-2.84
cg11660018*	PRSS23	protease. serine. 23	5.08E-20	7.46E-16	43.83	47.97	-4.14
cg04885881*			8.45E-20	1.2E-15	31.14	35.38	-4.23
cg11902777	AHRR	aryl-hydrocarbon receptor repressor	2.03E-19	2.78E-15	2.77	3.78	-1.00
cg27241845*			3.98E-18	5.28E-14	52.99	57.14	-4.15
cg24996979*	C14orf43	chrom 10 open reading frame 43	1.61E-17	2.06E-13	16.8	18.6	-1.79
cg03707168	PPP1R15A	protein phosphatase 1. regulatory subunit 15A	2.03E-17	2.53E-13	18.9	21.8	-2.90
cg23916896	AHRR	aryl-hydrocarbon receptor repressor	2.11E-17	2.56E-13	13.82	17.69	-3.87
cg11207515	CNTNAP2	contactin associated protein-like 2	5.26E-17	6.18E-13	34.98	30.15	4.83
cg25189904*	GNG12	guanine nucleotide binding protein (G protein). gamma 12	1.23E-16	1.4E-12	36.65	43.32	-6.67
cg04551776	AHRR	aryl-hydrocarbon receptor repressor	1E-15	1.11E-11	69.69	72.48	-2.79
cg01692968*			2.89E-15	3.13E-11	21.86	24.7	-2.84
cg07339236*	ATP9A	ATPase. class II. type 9A	3.11E-15	3.27E-11	7.46	9.06	-1.60
cg23771366*	PRSS23	protease. serine. 23	4.27E-15	4.39E-11	38.23	41.6	-3.37
cg03450842*	ZMIZ1	zinc finger. MIZ-type containing 1	9.29E-15	9.31E-11	54.63	57.26	-2.62
cg16145216	HIVEP3	human immunodeficiency virus type I enhancer binding protein 3	1.34E-14	1.31E-10	29.74	25.74	3.99

cg17287155	AHRR	aryl-hydrocarbon receptor repressor	1.91E-14	1.83E-10	81.7	84.66	-2.95
cg12876356	GFI1	growth factor independent 1 transcription repressor	2.09E-14	1.93E-10	65.52	70.94	-5.42
cg00310412*	SEMA7A	semaphorin 7A. GPI membrane anchor (John Milton Hagen blood group)	2.11E-14	1.93E-10	44.26	47.18	-2.93
cg20295214	AVPR1B	arginine vasopressin receptor 1B	4E-14	3.57E-10	60.49	63.71	-3.21
cg03991871	AHRR	aryl-hydrocarbon receptor repressor	5.86E-14	5.12E-10	68	73.54	-5.54
cg04180046	MYO1G	myosin IG	8.49E-14	7.27E-10	40.98	36.4	4.58
cg12513616*			8.74E-14	7.33E-10	38.75	41.32	-2.57
cg02657160	CPOX	coproporphyrinogen oxidase	1.14E-13	9.4E-10	78.1	80.16	-2.06
cg00073090			1.51E-13	1.21E-09	33.42	35.8	-2.37
cg12806681	AHRR	aryl-hydrocarbon receptor repressor	2E-13	1.58E-09	71.51	75.24	-3.74
cg15542713	HIVEP3	human immunodeficiency virus type I enhancer binding protein 3	3.16E-13	2.45E-09	44.32	37.95	6.37
cg24049493	HIVEP3	human immunodeficiency virus type I enhancer binding protein 3	1.76E-12	1.34E-08	19.33	14.97	4.36
cg01257799	CXCR5	chemokine (C-X-C motif) receptor 5	1.79E-12	1.34E-08	12.74	13.77	-1.03
cg19089201	MYO1G	myosin IG	2.5E-12	1.84E-08	80.87	73.34	7.54
cg16611234*			2.66E-12	1.92E-08	23.68	26.75	-3.07
cg02532700	NCF4	neutrophil cytosolic factor 4. 40kDa	4E-12	2.83E-08	13.75	16.22	-2.48
cg02451831	KIAA0087	KIAA0087	4.14E-12	2.88E-08	67.21	69.4	-2.19
cg14624207	LRP5	low density lipoprotein receptor-related protein 5	4.44E-12	3.04E-08	47.33	49.65	-2.32
cg01731783	C14orf43	chrom 10 open reading frame 43	5.06E-12	3.41E-08	56.42	58.7	-2.28
cg26361535	ZC3H3	zinc finger CCCH-type containing 3	7.81E-12	5.18E-08	63.94	67.65	-3.71
cg17619755	VARS	valyl-tRNA synthetase	9.92E-12	6.47E-08	58.68	54.42	4.26
cg06635952	ANXA4	annexin A4	1.26E-11	8.06E-08	25.72	23.94	1.78
cg21733098*			1.32E-11	8.36E-08	56.21	62.27	-6.06
cg01901332*	ARRB1	arrestin. beta 1	1.35E-11	8.41E-08	60.04	62.99	-2.95
cg18754985	CLDND1	claudin domain containing 1	1.38E-11	8.47E-08	85.16	87.16	-2.00
cg11554391	AHRR	aryl-hydrocarbon receptor repressor	1.62E-11	9.8E-08	11.85	13.54	-1.69
cg23973524	CRTC1	CREB regulated transcription coactivator 1	2.16E-11	1.29E-07	53.44	49.59	3.85
cg15159987*	CPAMD8	C3 and PZP-like. alpha-2-macroglobulin domain containing 8	2.71E-11	1.58E-07	55.17	57.55	-2.38
cg23480021			2.73E-11	1.58E-07	46.99	37.05	9.93
cg18146737	GFI1	growth factor independent 1 transcription repressor	3.23E-11	1.84E-07	55.74	71.1	-15.36
cg12075928	PTK2	protein tyrosine kinase 2	3.38E-11	1.9E-07	39.98	43.14	-3.16
cg09099830*	ITGAL	integrin. alpha L (antigen CD11A (p180). lymphocyte function-associated antigen 1; alpha polypeptide)	3.67E-11	2.04E-07	47.3	50.32	-3.01
cg20059012*	RARG	retinoic acid receptor. gamma	4.52E-11	2.48E-07	2.49	3.72	-1.23
cg06235438	ITGAL	integrin. alpha L (antigen CD11A (p180). lymphocyte function-associated antigen 1; alpha polypeptide)	4.9E-11	2.65E-07	65.81	68.21	-2.40
cg10750182	C10orf105	chrom 10 open reading frame 105	5.11E-11	2.69E-07	50.41	52.27	-1.85
cg18316974	GFI1	growth factor independent 1 transcription repressor	5.15E-11	2.69E-07	72.33	81.38	-9.05
cg13751113	AMICA1	adhesion molecule. interacts with CXADR antigen 1	5.16E-11	2.69E-07	14.29	15.61	-1.32
cg13039251	PDZD2	PDZ domain containing 2	5.23E-11	2.69E-07	68.79	64.09	4.70
cg07826859	MYO1G	myosin IG	6.48E-11	3.29E-07	50.64	52.89	-2.25
cg24090911	AHRR	aryl-hydrocarbon receptor repressor	1.48E-10	7.42E-07	57.96	61.43	-3.47
cg21473814	CRTC1	CREB regulated transcription coactivator 1	1.99E-10	9.86E-07	63.46	60.3	3.16
cg12593793			2.16E-10	1.06E-06	22.81	24.49	-1.68
cg11094248	RARA	retinoic acid receptor. alpha	2.25E-10	1.08E-06	10.64	11.57	-0.93

cg07202214	LRRC32	leucine rich repeat containing 32	2.26E-10	1.08E-06	22.68	24.58	-1.90
cg26271591	NFE2L2	nuclear factor. erythroid 2-like 2	2.74E-10	1.28E-06	27.5	30.92	-3.43
cg08709672*	AVPR1B	arginine vasopressin receptor 1B	2.75E-10	1.28E-06	53.33	55.98	-2.65
cg25292882			2.81E-10	1.3E-06	73.23	75.73	-2.50
cg25909396	PRKCA	protein kinase C. alpha	4.4E-10	2.01E-06	73.6	75.84	-2.25
cg20533899	LRRC32	leucine rich repeat containing 32	4.72E-10	2.13E-06	17.9	19.38	-1.48
cg03274391			5.1E-10	2.28E-06	46.53	38.9	7.63
cg19827923	GPR55	G protein-coupled receptor 55	5.77E-10	2.55E-06	73.71	75.44	-1.73
cg24908166	TERT	telomerase reverse transcriptase	6.35E-10	2.78E-06	82.6	85.71	-3.12
cg10874644			6.43E-10	2.78E-06	58.09	53.9	4.19
cg01554474	RAG1AP1	Solute carrier family 50 (sugar efflux transporter). member 1	6.8E-10	2.91E-06	20.42	22.49	-2.07
cg21393163			7.1E-10	3.01E-06	7.72	9.14	-1.43
cg13193840			7.31E-10	3.06E-06	3.93	4.64	-0.71
ch.1.171672612F			8.58E-10	3.56E-06	3.23	3.66	-0.43
cg13985437	LRRC32	leucine rich repeat containing 32	8.8E-10	3.62E-06	22.33	24.27	-1.93
cg11071448	SYT2	synaptotagmin II	9.51E-10	3.87E-06	39.04	41.57	-2.53
cg16391678	ITGAL	integrin. alpha L (antigen CD11A (p180). lymphocyte function-associated antigen 1; alpha polypeptide)	1.39E-09	5.61E-06	50.75	53.63	-2.88
cg16047567	DHRS3	dehydrogenase/reductase (SDR family) member 3	1.44E-09	5.73E-06	25.22	27.38	-2.16
cg01899089	AHRR	aryl-hydrocarbon receptor repressor	1.59E-09	6.28E-06	44.79	47.07	-2.28
cg20886049*	TSKU	tsukushi. small leucine rich proteoglycan	1.69E-09	6.63E-06	65.01	67.43	-2.42
cg19427338			1.73E-09	6.71E-06	73.73	71.1	2.63
cg04018738	VARS	valyl-tRNA synthetase	1.85E-09	7.1E-06	77.04	72.52	4.52
cg05603985	SKI	solute carrier family 24 (sodium/potassium/calcium exchanger). member 3	2.04E-09	7.77E-06	22.99	24.42	-1.43
cg19589396			2.07E-09	7.82E-06	55.16	58.07	-2.90
cg24540678			2.16E-09	8.07E-06	15.6	16.76	-1.16
cg13668129	HNRNPUL1	heterogeneous nuclear ribonucleoprotein U-like 1	2.37E-09	8.79E-06	18.85	19.97	-1.12
cg26963277	KCNQ1OT1	KCNQ1 opposite strand/antisense transcript 1 (non-protein coding)	3.25E-09	1.19E-05	82.42	85.17	-2.75
cg20244340	SLC24A3	Solute Carrier Family 24 Member 3	3.33E-09	1.21E-05	22.55	24.99	-2.44
cg19940644			3.36E-09	1.21E-05	27.21	30.21	-3.00
cg23351584	PRSS23	protease. serine. 23	3.88E-09	1.39E-05	9.46	10.33	-0.86
cg19713851	ALPP	alkaline phosphatase. placental	5.44E-09	1.93E-05	30.96	36.28	-5.33
cg13916835	SMG6	SMG6 nonsense mediated mRNA decay factor	6.04E-09	2.12E-05	63.02	66.12	-3.11
cg19614811	GPR15	G protein-coupled receptor 15	6.22E-09	2.16E-05	72.39	77.93	-5.54
cg26118759	CD58	CD58 molecule	7.27E-09	2.51E-05	3.44	3.98	-0.54
cg07251887	LOC100130		7.41E-09	2.54E-05	38.55	40.62	-2.07
cg13038618			8.01E-09	2.72E-05	44.72	46.77	-2.05
cg06007201	FAM38A	coagulation factor II (thrombin) receptor-like 3	8.29E-09	2.79E-05	5.75	6.5	-0.76
cg03604011	AHRR	aryl-hydrocarbon receptor repressor	9.29E-09	3.10E-5	4.17	3.37	0.80
cg09022230	TNRC18	trinucleotide repeat containing 18	9.36E-09	3.10E-5	61.93	64.42	-2.49
cg21446172	CAPN8	calpain 8	9.58E-09	3.15E-05	67.25	69.46	-2.21
cg08972170	C7orf41	chrom 7 open reading frame 41	9.69E-09	3.16E-05	46.29	43.2	3.09
cg26856289	SFRS13A	SKI proto-oncogene	9.81E-09	3.17E-05	23.22	24.75	-1.52
cg17372101	CNTNAP2	contactin associated protein-like 2	1.16E-08	3.74E-05	44.87	42.25	2.62
cg21188533	CACNA1D	calcium channel. voltage-dependent. L type. alpha 1D subunit	1.24E-08	3.96E-05	49.46	42.5	6.96
cg15693572			1.4E-08	4.43E-05	55.14	49.22	5.92
cg13633560	LRRC32	leucine rich repeat containing 32	1.59E-08	4.99E-05	31.64	33.77	-2.13
cg24134897	TSPAN4	tetraspanin 4	1.64E-08	5.10E-5	70.6	64.81	5.79
cg11557553	AHRR	aryl-hydrocarbon receptor repressor	1.72E-08	5.32E-05	75.93	74.37	1.56
cg26707709	SNED1	sushi. nidogen and EGF-like domains 1	1.78E-08	5.45E-05	10.31	7.76	2.55

cg23126342	PCDH9	protocadherin 9	1.85E-08	5.64E-05	45.46	40.89	4.58
cg05875421	GPR68	G protein-coupled receptor 68	1.97E-08	5.96E-05	9.83	10.87	-1.04
cg10814005	GPR68	G protein-coupled receptor 68	2E-08	5.97E-05	9.79	11.09	-1.31
cg06171420		metastasis suppressor 1	2.02E-08	5.97E-05	27.34	28.86	-1.52
cg24838345	MTSS1		2.02E-08	5.97E-05	63.45	67.25	-3.8
cg13185177	GP5	glycoprotein V (platelet)	2.12E-08	6.23E-05	41.87	38.93	2.94
cg26718213	SNED1	sushi. nidogen and EGF-like domains 1	2.24E-08	6.54E-05	23.27	18.44	4.83
cg12423733	MAS1L	MAS1 proto-oncogene like. G protein-coupled receptor	2.68E-08	7.75E-05	14.25	12.26	1.98
cg26764244	GNG12	guanine nucleotide binding protein (G protein). gamma 12	2.82E-08	8.11E-05	14.15	16.27	-2.12
cg11231349	NOS1AP	nitric oxide synthase 1 (neuronal) adaptor protein	2.92E-08	8.33E-05	63.18	66.66	-3.49
cg06106428	ARHGAP20	Rho GTPase activating protein 20	3.06E-08	8.68E-05	46.3	43.32	2.98
cg09662411	GFI1	growth factor independent 1 transcription repressor	3.17E-08	8.92E-05	60.31	63.76	-3.44
cg04517044	SMARCD3	SWI/SNF related. matrix associated. actin dependent regulator of chromatin. subfamily d. member 3	3.35E-08	9.34E-05	51.48	54.09	-2.61
cg05284742*	ITPK1	inositol-tetrakisphosphate 1-kinase	3.36E-08	9.34E-05	60.72	62.62	-1.90
cg13127741	COMMD7	COMM domain containing 7	3.39E-08	9.35E-05	40.85	42.87	-2.02
cg08595501	IQGAP2	IQ motif containing GTPase activating protein 2	3.88E-08	1.06E-04	55.64	58.65	-3.01
cg25560398	ECEL1P2	endothelin converting enzyme-like 1. pseudogene 2	3.89E-08	1.06E-04	55.99	57.58	-1.59
cg00336149	CACNA1D	calcium channel. voltage-dependent. L type. alpha 1D subunit	4.31E-08	1.16E-04	32.85	29.51	3.34
cg07986378	ETV6	ets variant 6	4.44E-08	1.19E-4	42.21	45.49	-3.29
cg13500388	CBFB	aryl-hydrocarbon receptor repressor	4.7E-08	1.25E-04	44.21	46.13	-1.93
cg16219322	AHRR	core-binding factor. beta subunit	4.7E-08	1.25E-04	77.9	79.16	-1.26
cg01442064	EVC	Ellis van Creveld syndrome	4.89E-08	1.28E-04	22.4	23.95	-1.55
cg06648759			4.89E-08	1.28E-04	51.91	49.81	2.10
cg12729894	HCCA2(MOB2)	MOB kinase activator 2	5.32E-08	1.38E-04	69.74	71.4	-1.66
cg27467282			5.38E-08	1.39E-04	76.55	74.59	1.96
cg04761231	RPL35	ribosomal protein L35	5.53E-08	1.42E-04	23.39	24.6	-1.21
cg13641317			5.86E-08	1.49E-04	21.15	19.24	1.91
cg07178945	FGF23	fibroblast growth factor 23	5.95E-08	1.51E-04	33.05	30.94	2.11
cg07151117	DUSP4	dual specificity phosphatase 4	6.21E-08	1.57E-04	11.58	12.45	-0.87
cg15022400	TRIM69	tripartite motif containing 69	6.36E-08	1.58E-04	13.76	15.23	-1.47
cg07465627	STXBP4	syntaxin binding protein 4	6.6E-08	1.63E-04	29.02	30.7	-1.68
ch.1.839062R	RUNX3	runt-related transcription factor 3	7.01E-08	1.73E-04	4.14	4.66	-0.53
cg26242531	ZFYVE21	zinc finger. FYVE domain containing 21	7.41E-08	1.81E-04	35.97	34.11	1.85
cg18660898	CDC42SE1	CDC42 small effector 1	7.54E-08	1.83E-04	7.01	7.9	-0.89
cg03188382	ALPP	alkaline phosphatase. placental	7.61E-08	1.84E-04	35.86	37.85	-1.99
cg14569771			7.75E-08	1.86E-04	44.42	42.06	2.36
cg04368724	VARS	valyl-tRNA synthetase	8.04E-08	1.92E-04	73.8	71.11	2.69
cg00501876	CSRNP1	cysteine-serine-rich nuclear protein 1	8.94E-08	2.12E-04	54.5	56.49	-2.00
cg05302489	VARS	valyl-tRNA synthetase	8.98E-08	2.12E-04	70.5	67.52	2.98
cg14420519			9.12E-08	2.14E-04	71.95	73.43	-1.48
cg07123182	KCNQ1OT1	KCNQ1 opposite strand/antisense transcript 1 (non-protein coding)	9.21E-08	2.15E-04	80.86	84.22	-3.36
cg23657179	C10orf41	chrom 10 open reading frame 43	9.38E-08	2.18E-04	38.58	43.29	-4.71
cg21618017	RILPL1	Rab interacting lysosomal protein-like 1	9.55E-08	2.20E-04	5.38	5.83	-0.45
cg13787850			9.7E-08	2.23E-04	35.24	37.5	-2.26
cg10919522	C14orf43	chrom 10 open reading frame 43	1.01E-07	2.31E-04	17.88	19.6	-1.72
cg16398761	C14orf43	chrom 10 open reading frame 43	1.06E-07	2.42E-04	1.86	2.19	-0.34
cg25004427	AHRR	aryl-hydrocarbon receptor repressor	1.09E-07	2.45E-04	78.74	76.76	1.99
cg22403782	ALPP	alkaline phosphatase. placental	1.09E-07	2.45E-04	27.33	30.42	-3.09
cg05969150	ZFHX3	zinc finger homeobox 3	1.11E-07	2.46E-04	34.95	33.58	1.37

cg16519923	ITGAL	integrin. alpha L (antigen CD11A (p180). lymphocyte function-associated antigen 1; alpha polypeptide)	1.21E-07	2.66E-04	56.07	58.45	-2.38
cg14901243			1.21E-07	2.66E-04	30.42	32.46	-2.02

Supplementary Table S3: CpG sites and associated genes differentially methylated in former smokers (FDR<0.05)

CpG site	gene	gene name	raw p value	FDR
cg05951221			1.76E-18	7.23E-13
cg21566642			1.22E-16	2.50E-11
cg06644428			7.04E-15	9.65E-10
cg05575921	AHRR	aryl-hydrocarbon receptor repressor	2.64E-12	2.72E-07
cg19859270	GPR15	G protein-coupled receptor 15	7.21E-12	5.93E-07
cg01940273			1.2E-11	8.24E-07
cg03636183	F2RL3	coagulation factor II (thrombin) receptor-like 3	1.7E-11	9.95E-07
cg13193840			1.22E-08	6.27E-04
cg03329539			1.65E-08	7.53E-04
cg03707168	PPP1R15A	protein phosphatase 1. regulatory subunit 15A	7.62E-08	3.13E-03
cg07525144	PRDM6		3.2E-07	1.19E-02
cg08233811	FLJ22536		5.62E-07	1.93E-02
cg05233390	CETN3		1.34E-06	4.24E-02
cg23351584	PRSS23	protease. serine. 23	1.46E-06	4.29E-02
cg12458913			1.58E-06	4.34E-02
cg23279756			1.71E-06	4.34E-02
cg16398451	POU6F1		1.96E-06	4.74E-02

Supplementary Table S4: miRNAs differentially expressed in smokers (FDR<0.10)

miRNA name	raw p value	FDR	foldchange expression
miR-6090	6.16E-06	3.40E-03	1.39
miR-4466	6.00E-05	1.11E-02	1.25
miR-3960	6.01E-05	1.11E-02	1.42
miR-4763-3p	1.00E-04	1.81E-02	1.22
miR-1207-5p	3.00E-04	2.41E-02	1.50
miR-135a-3p	3.00E-04	2.41E-02	1.21
miR-4281	3.00E-04	2.41E-02	1.32
miR-4270	7.00E-04	4.59E-02	1.20
miR-4687-3p	1.00E-03	6.22E-02	1.22
miR-20b-5p	1.30E-03	6.74E-02	0.81
miR-4516	1.30E-03	6.74E-02	1.43
miR-139-3p	1.50E-03	6.79E-02	1.27
miR-1225-5p	2.20E-03	8.13E-02	1.21
miR-4433-5p	2.50E-03	8.13E-02	1.45
miR-151a-5p	2.60E-03	8.13E-02	0.83
miR-2355-5p	2.60E-03	8.13E-02	1.60
miR-3648	2.70E-03	8.13E-02	1.88
let-7f-5p	3.10E-03	8.13E-02	0.84
miR-1537	3.10E-03	8.13E-02	1.67
miR-3196	3.10E-03	8.13E-02	1.16
miR-1234-5p	3.20E-03	8.13E-02	1.21
miR-4739	3.30E-03	8.13E-02	1.27
miR-193b-3p	3.40E-03	8.13E-02	1.52
miR-20a-5p	3.70E-03	8.61E-02	0.85
miR-3162-5p	4.20E-03	9.02E-02	1.18
miR-3665	4.20E-03	9.02E-02	1.26
miR-151b	4.60E-03	9.15E-02	0.83
miR-98-5p	4.90E-03	9.15E-02	0.77
miR-144-5p	5.00E-03	9.15E-02	0.67
let-7a-5p	5.10E-03	9.15E-02	1.24
miR-1229-5p	5.10E-03	9.15E-02	0.86
miR-937-5p	5.30E-03	9.18E-02	1.16
miR-4651	5.50E-03	9.21E-02	1.46
miR-4271	6.00E-03	9.84E-02	1.21

Supplementary Table S5: Full list of disease terms obtained using the different lists of smoking-modified genes (4 terms relating to experimentally induced conditions have been removed); Bonferoni-corrected p<0.05;

e: expression profile; m: CpG methylation profile; hubs: hub genes listed in Table 3; bold characters indicate the input list resulting in the lowest p-value

disease category	disease name	disease ID	lowest corrected p-value	input list#			
				e	m	e+m	hubs
Blood disease	Blood Coagulation Disorders	MESH:D001778	3.14E-03	+			
	Coagulation Protein Disorders	MESH:D020147	9.67E-03	+			
	Hematologic Diseases	MESH:D006402	1.01E-02			+	
Blood disease|Genetic disease (inborn)	Activated Protein C Resistance	MESH:D020016	2.53E-04	**+**		+	
Cancer	Neoplasms	MESH:D009369	2.92E-26	+	+	**+**	+
	Neoplasms by Site	MESH:D009371	2.26E-20	+	+	**+**	+
	Neoplasms by Histologic Type	MESH:D009370	1.56E-18	+	+	**+**	+
	Neoplasms. Glandular and Epithelial	MESH:D009375	1.05E-11		+	**+**	+
	Thoracic Neoplasms	MESH:D013899	2.32E-11	+	+	+	**+**
	Carcinoma	MESH:D002277	3.91E-10	+	+	+	**+**
	Head and Neck Neoplasms	MESH:D006258	3.51E-08		+	**+**	
	Adenocarcinoma	MESH:D000230	3.62E-08	+	+	**+**	+
	Carcinoma. Squamous Cell	MESH:D002294	3.75E-08		+	+	**+**
	Neoplasms. Squamous Cell	MESH:D018307	4.80E-08	+	+	+	**+**
	Neoplasms. Germ Cell and Embryonal	MESH:D009373	3.63E-05	+	+	**+**	
	Leukemia. Myeloid. Acute	MESH:D015470	1.26E-04		+	**+**	
	Neoplasms. Nerve Tissue	MESH:D009380	1.65E-04	+		**+**	
	Neuroectodermal Tumors	MESH:D017599	2.98E-04	+		**+**	
	Leukemia. Myeloid	MESH:D007951	7.46E-04		+	**+**	
	Leukemia	MESH:D007938	9.16E-04		+	**+**	
	Carcinoma. Transitional Cell	MESH:D002295	1.06E-03				+
	Neoplasms. Neuroepithelial	MESH:D018302	3.86E-03		+	**+**	
	Digestive System Neoplasms	MESH:D004067	2.39E-14	+	+	**+**	+
	Gastrointestinal Neoplasms	MESH:D005770	3.92E-10	+	+	**+**	+
	Intestinal Neoplasms	MESH:D007414	1.33E-07			+	**+**
	Colonic Neoplasms	MESH:D003110	5.08E-07				+
	Colorectal Neoplasms	MESH:D015179	4.58E-06			+	**+**
	Esophageal Neoplasms	MESH:D004938	4.24E-05		+	**+**	
	Liver Neoplasms	MESH:D008113	4.98E-05		+	**+**	+
	Carcinoma. Hepatocellular	MESH:D006528	2.98E-03		+	**+**	
	Pancreatic Neoplasms	MESH:D010190	2.98E-02				+
	Endocrine Gland Neoplasms	MESH:D004701	2.66E-04	+		**+**	+
	Lymphoma	MESH:D008223	4.01E-02			+	
	Neoplastic Processes	MESH:D009385	8.69E-05	+		+	**+**
	Neoplasm Invasiveness	MESH:D009361	6.74E-04			+	**+**
	Lymphatic Metastasis	MESH:D008207	2.18E-02			+	
	Lung Neoplasms	MESH:D008175	1.78E-11	+	+	+	**+**
	Respiratory Tract Neoplasms	MESH:D012142	2.10E-11	+	+	+	**+**
	Carcinoma. Bronchogenic	MESH:D002283	1.19E-02			+	
	Bronchial Neoplasms	MESH:D001984	1.19E-02			+	
	Breast Neoplasms	MESH:D001943	1.03E-05	+	+	**+**	+
	Genital Neoplasms. Female	MESH:D005833	1.53E-05	+		**+**	+
	Uterine Neoplasms	MESH:D014594	1.38E-03		+	**+**	
	Urogenital Neoplasms	MESH:D014565	1.40E-07		+	**+**	+
	Urinary Bladder Neoplasms	MESH:D001749	4.13E-04				+
	Urologic Neoplasms	MESH:D014571	2.40E-02				+
	Prostatic Neoplasms	MESH:D011471	6.83E-05		+	**+**	

	Genital Neoplasms. Male	MESH:D005834	1.76E-04		+	+	
Cardiovascular disease	Cardiovascular Diseases	MESH:D002318	1.19E-25		+	+	+
	Vascular Diseases	MESH:D014652	2.47E-21	+	+	+	+
	Heart Diseases	MESH:D006331	2.52E-14	+	+	+	+
	Myocardial Ischemia	MESH:D017202	2.18E-07	+	+	+	+
	Embolism and Thrombosis	MESH:D016769	1.40E-04	+		+	+
	Aortic Aneurysm	MESH:D001014	5.89E-04				+
	Aortic Diseases	MESH:D001018	5.89E-04				+
	Aneurysm	MESH:D000783	2.44E-03				+
	Thrombosis	MESH:D013927	2.74E-03	+		+	+
	Hypertension	MESH:D006973	7.14E-03	+		+	+
	Ventricular Outflow Obstruction	MESH:D014694	1.09E-02				+
	Coronary Disease	MESH:D003327	1.82E-02			+	
	Heart Failure	MESH:D006333	2.23E-02				+
	Coronary Artery Disease	MESH:D003324	2.48E-02			+	
	Myocardial Infarction	MESH:D009203	2.50E-02				+
	Arteriosclerosis	MESH:D001161	2.79E-02			+	
	Arterial Occlusive Diseases	MESH:D001157	4.77E-02			+	
Cardiovascular disease|Congenital abnormality	Cardiovascular Abnormalities	MESH:D018376	4.61E-02			+	
	Aortic Valve. Calcification of	MESH:C562942	2.34E-03				+
Cardiovascular disease|Nervous system disease	Cerebrovascular Disorders	MESH:D002561	5.40E-03			+	+
Cardiovascular disease|Pathology (anatomical condition)	Cardiomegaly	MESH:D006332	8.79E-09				+
	Reperfusion Injury	MESH:D015427	2.47E-03				+
Cardiovascular disease|Wounds and injuries	Vascular System Injuries	MESH:D057772	1.40E-02				+
Congenital abnormality	Congenital Abnormalities	MESH:D000013	4.03E-05		+	+	+
Congenital abnormality|Musculoskeletal disease	Craniofacial Abnormalities	MESH:D019465	1.15E-05		+	+	+
	Musculoskeletal Abnormalities	MESH:D009139	5.98E-05		+	+	+
Connective tissue disease	Connective Tissue Diseases	MESH:D003240	2.08E-05	+		+	
Connective tissue disease|Immune system disease|Musculoskeletal disease	Arthritis. Rheumatoid	MESH:D001172	3.04E-04	+		+	
Connective tissue disease|Musculoskeletal disease	Rheumatic Diseases	MESH:D012216	1.10E-05	+	+	+	
Digestive system disease	Digestive System Diseases	MESH:D004066	6.23E-23	+	+	+	+
	Gastrointestinal Diseases	MESH:D005767	1.95E-13	+	+	+	+
	Intestinal Diseases	MESH:D007410	1.44E-10	+		+	+
	Liver Diseases	MESH:D008107	3.94E-10	+	+	+	+
	Colonic Diseases	MESH:D003108	1.19E-08			+	+
	Liver Cirrhosis	MESH:D008103	7.41E-06				+
Digestive system disease|Substance-related disorder	Liver Cirrhosis. Alcoholic	MESH:D008104	3.54E-2				+
Digestive system disease	Gastroenteritis	MESH:D005759	1.19E-04				+
	Esophageal Diseases	MESH:D004935	1.30E-04		+	+	
	Colitis	MESH:D003092	1.58E-04				+
	Pancreatic Diseases	MESH:D010182	2.06E-03				+
	Stomach Diseases	MESH:D013272	3.02E-03	+		+	
	Crohn Disease	MESH:D003424	3.07E-03				+
	Rectal Diseases	MESH:D012002	2.56E-02				+
	Endocrine System Diseases	MESH:D004700	1.96E-08	+	+	+	+
Endocrine system disease|Metabolic disease	Diabetes Mellitus	MESH:D003920	1.67E-05	+		+	+
	Diabetes Mellitus. Type 2	MESH:D003924	2.90E-05	+		+	+
Endocrine system disease|Urogenital disease (female)	Ovarian Diseases	MESH:D010049	3.71E-02	+			
Eye disease	Eye Diseases	MESH:D005128	4.96E-03				+
Genetic disease (inborn)	Genetic Diseases. Inborn	MESH:D030342	1.59E-04	+		+	+
Immune system disease	Immune System Diseases	MESH:D007154	1.93E-15	+	+	+	+
	Autoimmune Diseases	MESH:D001327	2.73E-10	+	+	+	
	Hypersensitivity	MESH:D006967	2.25E-06	+		+	
	Immunoproliferative Disorders	MESH:D007160	2.83E-02			+	

Immune system disease|Lymphatic disease	Lymphoproliferative Disorders	MESH:D008232	1.57E-03			+	
Immune system disease|Nervous system disease	Demyelinating Autoimmune Diseases. CNS	MESH:D020278	4.43E-02			+	
Immune system disease|Respiratory tract disease	Asthma	MESH:D001249	3.62E-03	+			
Immune system disease|Respiratory tract disease	Respiratory Hypersensitivity	MESH:D012130	1.62E-02	+			
	Glomerulonephritis. IGA	MESH:D005922	3.96E-04			+	
Lymphatic disease	Lymphatic Diseases	MESH:D008206	2.03E-02			+	
Mental disorder	Mental Disorders	MESH:D001523	6.19E-11		+	+	+
	Schizophrenia and Disorders with Psychotic Features	MESH:D019967	4.45E-03		+	+	
	Mental Disorders Diagnosed in Childhood	MESH:D019952	5.63E-03		+	+	
	Schizophrenia	MESH:D012559	1.64E-02			+	
Mental disorder|Substance-related disorder	Substance-Related Disorders	MESH:D019966	4.04E-03		+	+	
	Amphetamine-Related Disorders	MESH:D019969	1.45E-02			+	
Metabolic disease	Metabolic Diseases	MESH:D008659	1.62E-07	+	+	+	+
	Glucose Metabolism Disorders	MESH:D044882	2.36E-06	+		+	+
	Calcium Metabolism Disorders	MESH:D002128	2.77E-04			+	+
	Calcinosis	MESH:D002114	7.10E-03				+
	Hyperinsulinism	MESH:D006946	1.55E-02				+
Mouth disease	Stomatognathic Diseases	MESH:D009057	1.92E-05	+	+	+	
	Mouth Diseases	MESH:D009059	2.22E-04		+	+	
Musculoskeletal disease	Musculoskeletal Diseases	MESH:D009140	6.71E-12	+	+	+	+
	Arthritis	MESH:D001168	3.30E-06	+	+	+	+
	Joint Diseases	MESH:D007592	1.04E-05		+	+	
	Bone Diseases	MESH:D001847	2.32E-05		+	+	+
	Spinal Diseases	MESH:D013122	7.93E-03				+
	Osteoporosis	MESH:D010024	1.24E-02		+	+	+
	Bone Diseases. Metabolic	MESH:D001851	1.44E-02		+	+	+
Musculoskeletal disease|Skin disease	Arthritis. Psoriatic	MESH:D015535	1.40E-02				+
Nervous system disease	Nervous System Diseases	MESH:D009422	5.49E-15	+	+	+	+
	Central Nervous System Diseases	MESH:D002493	2.02E-06		+	+	+
	Brain Diseases	MESH:D001927	3.32E-06	+	+	+	+
	Epilepsy	MESH:D004827	9.94E-05				+
	Neuromuscular Diseases	MESH:D009468	9.16E-04		+	+	+
	Neurotoxicity Syndromes	MESH:D020258	1.20E-03				+
	Demyelinating Diseases	MESH:D003711	2.32E-03			+	
	Basal Ganglia Diseases	MESH:D001480	3.12E-03				+
	Movement Disorders	MESH:D009069	1.01E-02				+
	Status Epilepticus	MESH:D013226	1.36E-02				+
	Heavy Metal Poisoning. Nervous System	MESH:D020260	1.49E-02				+
	Manganese Poisoning	MESH:D020149	1.75E-02				+
	Parkinson Disease	MESH:D010300	1.99E-02				+
	Neurodegenerative Diseases	MESH:D019636	3.90E-02				+
	Parkinsonian Disorders	MESH:D020734	4.00E-02				+
Nervous system disease|Signs and symptoms	Neurologic Manifestations	MESH:D009461	2.31E-04		+	+	+
	Pain	MESH:D010146	8.50E-04			+	+
	Hyperalgesia	MESH:D006930	1.69E-02				+
	Somatosensory Disorders	MESH:D020886	1.91E-02				+
Nutrition disorder	Overnutrition	MESH:D044343	5.54E-03				+
	Nutrition Disorders	MESH:D009748	1.64E-02				+
Nutrition disorder|Signs and symptoms	Obesity	MESH:D009765	5.54E-03				+
Occupational disease|Respiratory tract disease	Berylliosis	MESH:D001607	4.01E-02	+			
Pathology (anatomical condition)	Pathological Conditions. Anatomical	MESH:D020763	1.41E-11	+		+	+
	Hypertrophy	MESH:D006984	1.97E-05				+
	Ventricular Remodeling	MESH:D020257	3.91E-03				+
Pathology (process)	Pathologic Processes	MESH:D010335	6.65E-13	+	+	+	+
	Hyperplasia	MESH:D006965	5.89E-04			+	+

	Postoperative Complications	MESH:D011183	3.88E-03				+
	Hemorrhage	MESH:D006470	1.69E-02				+
	Fibrosis	MESH:D005355	1.76E-02				+
	Gliosis	MESH:D005911	4.66E-02				+
Respiratory tract disease	Respiratory Tract Diseases	MESH:D012140	1.67E-14	+	+	+	+
	Lung Diseases	MESH:D008171	7.05E-14	+	+	+	+
	Bronchial Diseases	MESH:D001982	1.75E-03	+			
	Lung Diseases. Obstructive	MESH:D008173	9.63E-03	+			
Respiratory tract disease|Wounds and injuries	Lung Injury	MESH:D055370	2.23E-02	+			
Signs and symptoms	Signs and Symptoms	MESH:D012816	3.32E-08		+	+	+
	Overweight	MESH:D050177	5.14E-03				+
	Body Weight	MESH:D001835	3.55E-02				+
Skin disease	Skin Diseases	MESH:D012871	1.68E-09		+	+	+
	Breast Diseases	MESH:D001941	6.95E-07		+	+	+
Urogenital disease (female)	Female Urogenital Diseases	MESH:D052776	3.62E-17	+	+	+	+
	Genital Diseases. Female	MESH:D005831	5.77E-07	+	+	+	+
	Uterine Diseases	MESH:D014591	3.35E-04		+	+	
	Adnexal Diseases	MESH:D000291	3.71E-02	+			
Urogenital disease (female)|Urogenital disease (male)	Urologic Diseases	MESH:D014570	4.73E-10	+	+	+	+
	Kidney Diseases	MESH:D007674	6.37E-08	+	+	+	+
	Glomerulonephritis	MESH:D005921	2.21E-05	+		+	+
	Nephritis	MESH:D009393	7.39E-05	+		+	+
	Urinary Bladder Diseases	MESH:D001745	9.64E-04				+
Urogenital disease (male)	Male Urogenital Diseases	MESH:D052801	1.42E-13	+	+	+	+
	Prostatic Diseases	MESH:D011469	7.96E-05		+	+	
	Genital Diseases. Male	MESH:D005832	2.10E-04		+	+	+
	Female Urogenital Diseases and Pregnancy Complications	MESH:D005261	3.67E-19	+	+	+	+
	Pathological Conditions. Signs and Symptoms	MESH:D013568	2.94E-17	+	+	+	+
	Skin and Connective Tissue Diseases	MESH:D017437	1.15E-13	+	+	+	+
	Chemically-Induced Disorders	MESH:D064419	1.05E-08	+	+	+	+
	Congenital. Hereditary. and Neonatal Diseases and Abnormalities	MESH:D009358	2.87E-08	+	+	+	+
	Nutritional and Metabolic Diseases	MESH:D009750	5.18E-08	+	+	+	+
	Hemic and Lymphatic Diseases	MESH:D006425	9.52E-06	+	+	+	
	Poisoning	MESH:D011041	3.69E-05			+	+
	Drug-Related Side Effects and Adverse Reactions	MESH:D064420	2.41E-03	+		+	

Supplementary Table S6: Pathways associated with the combined sets of DEGs and DMGs as derived in ConsensusPathDB; (FDR<0.05)

pathway	source	FDR
TGF-β signaling		
TGF Beta Signaling Pathway	Wikipathways	1.16E-03
Downregulation of TGF-beta receptor signaling	Reactome	5.17E-03
Loss of Function of SMAD2/3 in Cancer	Reactome	5.17E-03
Loss of Function of SMAD4 in Cancer	Reactome	5.17E-03
Loss of Function of TGFBR1 in Cancer	Reactome	5.17E-03
Loss of Function of TGFBR2 in Cancer	Reactome	5.17E-03
Signaling by TGF-beta Receptor Complex	Reactome	5.17E-03
Signaling by TGF-beta Receptor Complex in Cancer	Reactome	5.17E-03
SMAD2/3 MH2 Domain Mutants in Cancer	Reactome	5.17E-03
SMAD2/3 Phosphorylation Motif Mutants in Cancer	Reactome	5.17E-03
SMAD4 MH2 Domain Mutants in Cancer	Reactome	5.17E-03
TGFBR1 KD Mutants in Cancer	Reactome	5.17E-03
TGFBR1 LBD Mutants in Cancer	Reactome	5.17E-03
TGFBR2 Kinase Domain Mutants in Cancer	Reactome	5.17E-03
TGFBR2 MSI Frameshift Mutants in Cancer	Reactome	5.17E-03
TGF-beta receptor signaling activates SMADs	Reactome	1.02E-02
Regulation of nuclear SMAD2/3 signaling	PID	1.89E-02
TGF_beta_Receptor	NetPath	3.08E-02
Transcriptional activity of SMAD2/SMAD3:SMAD4 heterotrimer	Reactome	3.90E-02
Thrombin signaling / blood coagulation		
Platelet activation. signaling and aggregation	Reactome	1.16E-03
Hemostasis	Reactome	3.53E-03
Platelet Adhesion to exposed collagen	Reactome	1.72E-02
Formation of Fibrin Clot (Clotting Cascade)	Reactome	2.24E-02
extrinsic prothrombin activation pathway	BioCarta	3.08E-02
Platelet Aggregation (Plug Formation)	Reactome	4.36E-02
Cell-cell and cell-matrix interactions		
Adherens junction - Homo sapiens (human)	KEGG	1.16E-03
Focal Adhesion	Wikipathways	3.53E-03
Cell surface interactions at the vascular wall	Reactome	2.04E-02
Other		
Inflammatory bowel disease (IBD) - Homo sapiens (human)	KEGG	3.53E-03
Hematopoietic cell lineage - Homo sapiens (human)	KEGG	4.72E-03
HIF-1 signaling pathway - Homo sapiens (human)	KEGG	6.45E-03
Direct p53 effectors	PID	6.77E-03
Angiopoietin receptor Tie2-mediated signaling	PID	9.34E-03
HTLV-I infection - Homo sapiens (human)	KEGG	9.84E-03
Axon guidance - Homo sapiens (human)	KEGG	1.18E-02
EPHA-mediated growth cone collapse	Reactome	1.55E-02
Primary Focal Segmental Glomerulosclerosis FSGS	Wikipathways	1.83E-02
Arf6 signaling events	PID	1.89E-02
Axon guidance	Reactome	1.89E-02
Cell-Cell communication	Reactome	1.89E-02
DCC mediated attractive signaling	Reactome	1.89E-02
IL-3 Signaling Pathway	Wikipathways	1.89E-02
Integrated Pancreatic Cancer Pathway	Wikipathways	1.89E-02
Integrin-mediated Cell Adhesion	Wikipathways	1.89E-02
Jak-STAT signaling pathway - Homo sapiens (human)	KEGG	1.89E-02
Rap1 signaling pathway - Homo sapiens (human)	KEGG	1.89E-02
Signalling by NGF	Reactome	1.89E-02
Ras signaling pathway - Homo sapiens (human)	KEGG	2.11E-02
mcalpain and friends in cell motility	BioCarta	2.12E-02
CXCR4-mediated signaling events	PID	2.13E-02
ErbB Signaling Pathway	Wikipathways	2.21E-02
Interferon type I signaling pathways	Wikipathways	2.21E-02
Syndecan interactions	Reactome	2.21E-02

miR-targeted genes in muscle cell - TarBase	Wikipathways	2.23E-02
Allograft Rejection	Wikipathways	2.24E-02
Allograft rejection - Homo sapiens (human)	KEGG	2.24E-02
Common Pathway	Reactome	2.24E-02
Graft-versus-host disease - Homo sapiens (human)	KEGG	2.24E-02
Regulation of Complement cascade	Reactome	2.24E-02
AMPK Signaling	Wikipathways	2.28E-02
G alpha (i) signalling events	Reactome	2.40E-02
Type I diabetes mellitus - Homo sapiens (human)	KEGG	2.40E-02
Pancreatic cancer - Homo sapiens (human)	KEGG	2.45E-02
EGF-EGFR Signaling Pathway	Wikipathways	2.76E-02
Aryl Hydrocarbon Receptor	Wikipathways	2.91E-02
Developmental Biology	Reactome	2.91E-02
integrin signaling pathway	BioCarta	2.91E-02
PI3K-Akt signaling pathway - Homo sapiens (human)	KEGG	2.95E-02
granzyme a mediated apoptosis pathway	BioCarta	2.96E-02
Leishmaniasis - Homo sapiens (human)	KEGG	2.96E-02
Alternative complement activation	Reactome	3.08E-02
Autoimmune thyroid disease - Homo sapiens (human)	KEGG	3.08E-02
Complement and Coagulation Cascades	Wikipathways	3.08E-02
Complement cascade	Reactome	3.08E-02
IL12 signaling mediated by STAT4	PID	3.08E-02
Viral myocarditis - Homo sapiens (human)	KEGG	3.08E-02
il-2 receptor beta chain in t cell activation	BioCarta	3.14E-02
Pathways in cancer - Homo sapiens (human)	KEGG	3.38E-02
ErbB signaling pathway - Homo sapiens (human)	KEGG	3.40E-02
Plexin-D1 Signaling	PID	3.40E-02
regulation of bad phosphorylation	BioCarta	3.40E-02
Regulation of KIT signaling	Reactome	3.40E-02
cbl mediated ligand-induced downregulation of egf receptors pathway	BioCarta	3.63E-02
NGF signalling via TRKA from the plasma membrane	Reactome	3.84E-02
Activation of SMO	Reactome	3.87E-02
a6b1 and a6b4 Integrin signaling	PID	3.90E-02
Arrhythmogenic right ventricular cardiomyopathy	KEGG	3.90E-02
HIF-1-alpha transcription factor network	PID	3.90E-02
IL4-mediated signaling events	PID	4.04E-02
Human Complement System	Wikipathways	4.28E-02
IL12-mediated signaling events	PID	4.28E-02
Signaling events mediated by focal adhesion kinase	PID	4.36E-02
ErbB1 downstream signaling	PID	4.54E-02
Endochondral Ossification	Wikipathways	4.71E-02
Type I hemidesmosome assembly	Reactome	4.91E-02
EPH-Ephrin signaling	Reactome	4.99E-02
Regulation of retinoblastoma protein	PID	4.99E-02

Supplementary Table S7: GO terms (Comparative Toxicogenomics database) related to the set of hub genes; the total number of GO terms significant at p<0.01 is 1028; here are shown the top 200 terms with level ≥ 4 on the GO tree

GO term name	ontology	highest GO level	Bonferoni-corrected p-value
cell surface receptor signaling pathway	Biological Process	4	1.35E-32
cellular response to organic substance	Biological Process	4	1.76E-31
regulation of apoptotic process	Biological Process	5	2.81E-30
regulation of programmed cell death	Biological Process	4	3.51E-30
intracellular signal transduction	Biological Process	4	9.57E-29
negative regulation of apoptotic process	Biological Process	5	1.40E-28
negative regulation of programmed cell death	Biological Process	4	1.86E-28
positive regulation of intracellular signal transduction	Biological Process	4	3.99E-27
apoptotic process	Biological Process	5	8.61E-27
positive regulation of cellular biosynthetic process	Biological Process	4	1.20E-26
programmed cell death	Biological Process	4	1.22E-26
regulation of protein metabolic process	Biological Process	4	1.51E-26
regulation of intracellular signal transduction	Biological Process	4	2.72E-24
regulation of cellular biosynthetic process	Biological Process	4	4.00E-23
cellular response to growth factor stimulus	Biological Process	5	5.78E-23
response to growth factor	Biological Process	4	9.82E-23
protein phosphorylation	Biological Process	6	1.53E-22
positive regulation of protein metabolic process	Biological Process	4	1.60E-22
positive regulation of macromolecule biosynthetic process	Biological Process	4	2.10E-22
cardiovascular system development	Biological Process	4	2.37E-22
circulatory system development	Biological Process	4	2.37E-22
epithelium development	Biological Process	4	4.13E-22
enzyme linked receptor protein signaling pathway	Biological Process	5	4.32E-22
positive regulation of nucleobase-containing compound metabolic process	Biological Process	4	7.57E-22
cell-type specific apoptotic process	Biological Process	6	1.42E-21
cell differentiation	Biological Process	4	1.89E-21
phosphate-containing compound metabolic process	Biological Process	4	2.49E-21
regulation of phosphate metabolic process	Biological Process	5	5.22E-21
regulation of phosphorus metabolic process	Biological Process	4	6.52E-21
positive regulation of phosphate metabolic process	Biological Process	5	2.51E-20
positive regulation of phosphorus metabolic process	Biological Process	4	2.51E-20
organic cyclic compound biosynthetic process	Biological Process	4	2.85E-20
cellular protein modification process	Biological Process	5	6.38E-20
protein modification process	Biological Process	4	6.38E-20
phosphorylation	Biological Process	5	1.59E-19
macromolecule modification	Biological Process	4	2.34E-19
heterocycle biosynthetic process	Biological Process	4	3.37E-19
positive regulation of nucleic acid-templated transcription	Biological Process	6	4.30E-19
positive regulation of transcription. DNA-templated	Biological Process	5	4.30E-19
cellular protein metabolic process	Biological Process	4	4.58E-19
regulation of nucleobase-containing compound metabolic process	Biological Process	4	4.82E-19
positive regulation of gene expression	Biological Process	4	4.83E-19
vasculature development	Biological Process	4	5.24E-19
cellular nitrogen compound biosynthetic process	Biological Process	4	5.48E-19
positive regulation of RNA biosynthetic process	Biological Process	5	7.05E-19
apoptotic signaling pathway	Biological Process	4	1.32E-18
positive regulation of RNA metabolic process	Biological Process	4	1.33E-18
regulation of gene expression	Biological Process	4	1.85E-18
nucleobase-containing compound biosynthetic process	Biological Process	4	5.40E-18
aromatic compound biosynthetic process	Biological Process	4	9.08E-18
protein kinase binding	Molecular Function	5	1.95E-17
transcription from RNA polymerase II promoter	Biological Process	6	3.25E-17
gene expression	Biological Process	4	3.54E-17
positive regulation of transcription from RNA polymerase II promoter	Biological Process	6	6.43E-17
regulation of cellular protein metabolic process	Biological Process	4	8.62E-17

regulation of transcription from RNA polymerase II promoter	Biological Process	6	9.61E-17
regulation of cellular macromolecule biosynthetic process	Biological Process	5	1.00E-16
kinase binding	Molecular Function	4	1.31E-16
positive regulation of phosphorylation	Biological Process	6	1.97E-16
regulation of protein phosphorylation	Biological Process	6	2.21E-16
regulation of macromolecule biosynthetic process	Biological Process	4	2.25E-16
vesicle-mediated transport	Biological Process	4	2.67E-16
regulation of phosphorylation	Biological Process	6	2.95E-16
positive regulation of protein phosphorylation	Biological Process	6	4.37E-16
regulation of protein modification process	Biological Process	5	8.27E-16
response to organic cyclic compound	Biological Process	4	8.30E-16
wound healing	Biological Process	4	1.06E-15
positive regulation of cellular protein metabolic process	Biological Process	4	1.91E-15
regulation of lipid metabolic process	Biological Process	4	2.53E-15
regulation of transcription. DNA-templated	Biological Process	5	4.36E-15
regulation of apoptotic signaling pathway	Biological Process	4	4.42E-15
MAPK cascade	Biological Process	4	4.83E-15
regulation of nucleic acid-templated transcription	Biological Process	6	5.26E-15
regulation of RNA biosynthetic process	Biological Process	5	6.14E-15
regulation of establishment of protein localization	Biological Process	4	6.24E-15
cellular macromolecule biosynthetic process	Biological Process	4	6.48E-15
cellular response to oxygen-containing compound	Biological Process	4	1.02E-14
regulation of RNA metabolic process	Biological Process	4	1.29E-14
positive regulation of MAPK cascade	Biological Process	5	1.34E-14
nucleic acid metabolic process	Biological Process	4	1.53E-14
macromolecule biosynthetic process	Biological Process	4	1.60E-14
extrinsic apoptotic signaling pathway	Biological Process	5	2.40E-14
transmembrane receptor protein serine/threonine kinase signaling pathway	Biological Process	6	2.51E-14
negative regulation of extrinsic apoptotic signaling pathway	Biological Process	5	2.51E-14
cellular response to transforming growth factor beta stimulus	Biological Process	4	2.68E-14
regulation of cell migration	Biological Process	4	2.75E-14
reproductive system development	Biological Process	4	2.97E-14
regulation of MAPK cascade	Biological Process	5	3.07E-14
transcription. DNA-templated	Biological Process	5	3.31E-14
response to lipid	Biological Process	4	3.48E-14
positive regulation of protein modification process	Biological Process	5	3.72E-14
positive regulation of programmed cell death	Biological Process	4	3.72E-14
nucleic acid-templated transcription	Biological Process	6	3.79E-14
protein transport	Biological Process	4	3.99E-14
RNA biosynthetic process	Biological Process	5	8.79E-14
negative regulation of nucleobase-containing compound metabolic process	Biological Process	4	2.25E-13
regulation of epithelial cell proliferation	Biological Process	4	2.96E-13
transforming growth factor beta receptor signaling pathway	Biological Process	5	3.41E-13
ameboidal-type cell migration	Biological Process	4	3.70E-13
negative regulation of apoptotic signaling pathway	Biological Process	4	4.85E-13
epithelial cell migration	Biological Process	5	5.13E-13
negative regulation of cellular biosynthetic process	Biological Process	4	5.91E-13
epithelium migration	Biological Process	4	6.09E-13
positive regulation of apoptotic process	Biological Process	5	1.29E-12
endocytosis	Biological Process	5	1.62E-12
inflammatory response	Biological Process	4	2.71E-12
organic substance transport	Biological Process	4	2.99E-12
RNA metabolic process	Biological Process	4	4.02E-12
morphogenesis of an epithelium	Biological Process	4	5.00E-12
regulation of extrinsic apoptotic signaling pathway	Biological Process	5	5.38E-12
nervous system development	Biological Process	4	6.51E-12
nucleus	Cellular Component	4	6.68E-12
regulation of cellular protein localization	Biological Process	4	9.35E-12
response to decreased oxygen levels	Biological Process	4	1.07E-11
negative regulation of transmembrane receptor protein serine/threonine kinase signaling pathway	Biological Process	4	1.26E-11
cytosol	Cellular Component	4	2.08E-11
ERK1 and ERK2 cascade	Biological Process	5	2.10E-11
intrinsic apoptotic signaling pathway	Biological Process	5	2.17E-11
regulation of transmembrane receptor protein serine/threonine kinase signaling pathway	Biological Process	4	2.48E-11
protein localization to nucleus	Biological Process	6	4.40E-11
neurogenesis	Biological Process	5	5.30E-11

regulation of protein serine/threonine kinase activity	Biological Process	7	6.33E-11
epithelial cell differentiation	Biological Process	5	6.54E-11
positive regulation of cell migration	Biological Process	4	6.62E-11
regulation of protein transport	Biological Process	4	7.66E-11
positive regulation of protein kinase B signaling	Biological Process	5	8.21E-11
regulation of kinase activity	Biological Process	5	9.72E-11
regulation of proteolysis	Biological Process	5	1.09E-10
protein kinase B signaling	Biological Process	5	1.34E-10
muscle tissue development	Biological Process	4	1.34E-10
digestive system development	Biological Process	4	1.34E-10
transmembrane receptor protein tyrosine kinase signaling pathway	Biological Process	6	1.39E-10
peptidyl-amino acid modification	Biological Process	6	1.47E-10
regulation of protein catabolic process	Biological Process	4	1.64E-10
chordate embryonic development	Biological Process	5	1.75E-10
embryo development ending in birth or egg hatching	Biological Process	4	1.98E-10
epidermal growth factor receptor signaling pathway	Biological Process	8	2.20E-10
negative regulation of protein metabolic process	Biological Process	4	2.43E-10
regulation of cell-cell adhesion	Biological Process	4	2.51E-10
protein catabolic process	Biological Process	4	2.54E-10
protein import into nucleus	Biological Process	4	2.61E-10
protein targeting to nucleus	Biological Process	4	2.61E-10
single-organism nuclear import	Biological Process	4	2.61E-10
negative regulation of gene expression	Biological Process	4	2.74E-10
ERBB signaling pathway	Biological Process	7	2.84E-10
nuclear import	Biological Process	7	3.09E-10
immune response-regulating signaling pathway	Biological Process	4	3.95E-10
generation of neurons	Biological Process	6	4.29E-10
response to nutrient levels	Biological Process	4	5.61E-10
regulation of cell development	Biological Process	4	5.69E-10
regulation of ERK1 and ERK2 cascade	Biological Process	6	7.41E-10
regulation of transferase activity	Biological Process	4	7.68E-10
regulation of hydrolase activity	Biological Process	4	8.69E-10
regulation of osteoblast differentiation	Biological Process	4	9.56E-10
negative regulation of cellular macromolecule biosynthetic process	Biological Process	5	1.07E-09
regulation of defense response	Biological Process	4	1.12E-09
regulation of protein kinase activity	Biological Process	6	1.17E-09
blood circulation	Biological Process	5	1.55E-09
regulation of mitotic cell cycle	Biological Process	4	1.55E-09
circulatory system process	Biological Process	4	1.64E-09
negative regulation of cellular protein metabolic process	Biological Process	4	1.75E-09
regulation of angiogenesis	Biological Process	4	1.94E-09
fibroblast growth factor receptor signaling pathway	Biological Process	5	2.03E-09
protein secretion	Biological Process	4	2.08E-09
mitotic cell cycle	Biological Process	4	2.18E-09
peptidyl-tyrosine phosphorylation	Biological Process	7	2.28E-09
regulation of protein kinase B signaling	Biological Process	5	2.32E-09
peptidyl-tyrosine modification	Biological Process	7	2.44E-09
osteoblast differentiation	Biological Process	4	2.57E-09
negative regulation of macromolecule biosynthetic process	Biological Process	4	2.70E-09
leukocyte cell-cell adhesion	Biological Process	4	3.07E-09
regulation of vasculature development	Biological Process	4	4.04E-09
negative regulation of transcription. DNA-templated	Biological Process	5	4.26E-09
cellular response to organic cyclic compound	Biological Process	5	4.27E-09
intracellular transport	Biological Process	4	4.48E-09
cellular response to fibroblast growth factor stimulus	Biological Process	4	5.24E-09
epithelial tube morphogenesis	Biological Process	4	5.49E-09
negative regulation of nucleic acid-templated transcription	Biological Process	6	6.53E-09
mammary gland development	Biological Process	4	6.57E-09
response to light stimulus	Biological Process	4	7.45E-09
central nervous system development	Biological Process	4	7.49E-09
negative regulation of RNA biosynthetic process	Biological Process	5	7.94E-09
positive regulation of protein kinase activity	Biological Process	6	8.62E-09
endothelial cell migration	Biological Process	6	8.87E-09
regulation of protein binding	Biological Process	4	9.40E-09
negative regulation of osteoblast differentiation	Biological Process	4	1.04E-08
negative regulation of transcription from RNA polymerase II promoter	Biological Process	6	1.27E-08
negative regulation of RNA metabolic process	Biological Process	4	1.30E-08

positive regulation of epithelial cell proliferation	Biological Process	4	1.40E-08
positive regulation of kinase activity	Biological Process	5	1.50E-08
cellular response to hormone stimulus	Biological Process	4	1.79E-08
response to steroid hormone	Biological Process	4	1.86E-08
platelet activation	Biological Process	4	2.01E-08
negative regulation of anoikis	Biological Process	6	2.10E-08
chemical homeostasis	Biological Process	4	2.38E-08
neuron death	Biological Process	4	2.87E-08
positive regulation of protein serine/threonine kinase activity	Biological Process	7	3.30E-08
nucleocytoplasmic transport	Biological Process	6	3.79E-08
nuclear transport	Biological Process	5	4.30E-08
neuron differentiation	Biological Process	5	4.31E-08

Supplementary Table S8: Smoking-induced changes in component genes of key pathways

pathway (database)	role in smoking-related disease / impact of tobacco smoke	no. of smoking-modified genes / total no. of genes	gene symbol	number of CpG sites significantly (FDR<0.05) modified (direction of methylation change in smokers)	direction of expression change of significan (FDR<0.10) DEGs in smokers	number of differentially expressed miRNAs associated with gene (direction of expression change in smokers)
Thrombin signaling through proteinase activated receptors (PARs) (Reactome)	plays a key role in blood coagulation and is important in cardiovascular diseases. while cigarette smoke impairs vasodilation and fibrinolysis mediated by thrombin-activated proteinase type-1 receptors. thus leading to enhancement of clot expansion and vessel occlusion[11]	9/32	ARRB1	1 (down)		
			ARRB2	1 (down)		
			F2R	1 (down)	down	4 (1 up. 3 down)
			F2RL3	3 (down)		
			GNG11		down	
			GNG12	5 (down)		
			GNG7	3 (down)		
			GNGT2		down	
			SRC	3 (up)		
Role of nicotinic acetylcholine receptors in the regulation of apoptosis (Biocarta)	involved in the regulation of apoptosis and may be important for carcinogenesis since nicotine blocks apoptosis in lung epithelial cells by activating the anti-apoptotic kinase AKT[12]	6/17	AKT1	2 (down)		
			FOXO3	1 (down)		
			MUSK	1 (down)		
			PTK2	1 (down)	down	3 (up)
			SRC	3 (up)		
			TERT	3 (down)		
Aryl hydrocarbon receptor pathway (Wikipathways)	controls a number of important biological processes. including the metabolism of tobacco smoke carcinogens. cell proliferation and apoptosis[13]	12/44	AHRR	27 (9 up. 18 down)	up	1 (up)
			CDKN1A	4 (down)		
			CYP1B1		up	2 (1 up. 1 down)
			E2F1	1 (down)		
			ESR1	1 (up)		
			KLF6	4 (down)		
			KRAS	1 (down)		
			NFE2L2	1 (down)		
			PLAGL1	2 (up)		
			RELA	1 (down)		
			SRC	3 (2 up. 1 down)		
			TNF	7 (down)		
GABAergic synapse (KEGG)	key players in the	17/90	ADCY9	2 (down)	down	4 (2 up. 2 down)

	regulation of the behavioural and biochemical alterations related to addiction to various drugs[14]. while in addition several lines of evidence suggest a role of this pathway in carcinogenesis[15]		CACNA1C	2 (down)		
			CACNA1D	4 (up)		
			GABBR1	1 (up)		
			GABRA2	1 (down)		
			GABRB1	1 (up)		
			GABRG2	1 (up)		
			GABRR2	1 (down)		
			GNAI2	1 (down)		
			GNG11		down	
			GNG12	5 (down)		
			GNG7	3 (down)		
			GNGT2		down	
			HAP1	1 (down)		
			PRKCA	3 (down)		
			SLC38A3	1 (down)		
			SMAD7		down	1 (down)
TGF-Beta pathway (Wikipathways)	plays an important role in cellular protection against the damaging effects of tobacco smoke[16] and it is notable that most of the hub genes involved in the corresponding signaling pathway are underexpressed in smokers	18/54	BAMBI	1 (down)		
			CREBBP	1 (down)		
			EGF	1 (up)		
			JUN	1 (up)		
			LTBP1	1 (down)	down	
			RUNX2	1 (down)		
			RUNX3	7 (down)	down	1 (down)
			SKI	6 (down)		
			SKIL	1 (down)		
			SMAD2	1 (down)		
			SMAD3	2 (down)		
			SMAD6	1 (down)		
			SRC	3 (up)		
			STAT3	2 (down)		
			TGFBR3		down	
			THBS1	1 (down)		
			TNF	14 (down)		
			ZNF423	1 (up)		
ErbB Signaling Pathway (Wikipathways)	controls many cellular responses. including cytoskeletal rearrangement. apoptosis and cell proliferation. while tobacco smoke can initiate pathway activation by activating metalloproteinases which cleave EGFR	15/54	CCND1	1 (down)		
			CDKN1A	4 (down)		
			EGF	1 (up)		
			ERBB2		1 (down)	4 (up)
			JUN	1 (up)		
			KRAS	1 (up)		
			NRG1	1 (down)		
			NRG2	4 (down)		
			PAK4	2 (down)		
			PIK3R5	1 (down)		
			PRKCA	3 (1 up. 2 down)		
			PTK2	1 (down)	2 (down)	3 (up)

	proligands[17]		SHC2	1 (up)		
			SRC	3 (2 up. 1 down)		
			STAT5A	3 (down)		

REFERENCES

1. Charlesworth, J.C. *et al.*, Transcriptomic epidemiology of smoking: The effect of smoking on gene expression in lymphocytes. *BMC Med. Genomics* **3:29,** doi: 10.1186/1755-8794-3-29 (2010).

2. Beineke, P. *et al.* A whole blood gene expression-based signature for smoking status. *BMC Med. Genomics* **5:58,** doi: 10.1186/1755-8794-5-58 (2012).

3. Verdugo, R. A. *et al*. Graphical modeling of gene expression in monocytes suggests molecular mechanisms explaining increased atherosclerosis in smokers. *PLoS One* **8**, e50888 (2013).

4. Wan, E. S. *et al.*, Cigarette smoking behaviors and time since quitting are associated with differential DNA methylation across the human genome. *Hum. Mol. Genet.* **21,** 3073-3082 (2012).

5. Shenker, I . M. S. *et al.*, Epigenome-wide association study in the European Prospective Investigation into Cancer and Nutrition (EPIC-Turin) identifies novel genetic loci associated with Smoking. *Hum. Mol. Genet.* **22,** 843-851 (2012).

6. Zeilinger, S. *et al*. tobacco smoking leads to extensive genome-wide changes in DNA methylation. *PLoS One* 8, e638l2 (2013).

7. Harlid, S., Xu, Z., Panduri, V., Sandier, D. P. & Taylor, J. A. CpG sites associated with cigarette smoking: analysis of epigenome-wide data from the Sister Study. *Environ. Health. Perspect.* 122, 673-678 (2014).

8. Elliott, H. R. *et al.* Differences in smoking associated DNA methylation patterns in South Asians and Europeans. Clin Epigenetics **6(1):4**, doi: 10.1186/1868-7083-6-4 (2014)

9. Monick, M. M., Beach, S. R, Plume, J., Sears, R., Gerrard, M., Brody, G. H. & Philibert, R. A. Coordinated changes in AHRR methylation in lymphoblasts and pulmonary macrophages from Smokers. *Am. J. Med. Genet. B Neuropsychiatr. Genet.* **1596,** 141-151 (2012).

10. Sun YV, Smith AK, Conneeiy KN, Chang Q, Li W, Lazarus A, Smith JA, Almli LM, Binder EB, Klengel T, Cross D, Turner ST, Ressler KJ, Kardia SL. (2013) Epigenomic association analysis identifies smoking-related DNA methylation sites in African Americans. Hum Genet. 2013 Sep;132(9):1027-37

11. Lang. N. N. *et al.* Marked impairment of protease-activated receptor type 1-mediated vasoditation and fibrinolysis in cigarette smokers: smoking. thrombin. and vascular responses in vivo. *J. Am. Coll. Cardiol.* **52.** 33-39 (2008).

12. West. K. A. *et al.* Rapid Akt activation by nicotine and a tobacco carcinogen modulates the phenotype of normal human airway epithelial cells. *J. Clin. Invest.* **111.** 81-90 (2003).

13. Feng. S.. Cao. Z. & Wang. X. Role of aryl hydrocarbon receptor in cancer. *Biochim Biophys Acta* **1836**. 197-210 (2013).

14. Varani. A. P. *et al.* Lack of GABAB receptors modifies behavioural and biochemical alterations induced by precipitated nicotine withdrawal. *Neuropharmacology* **90.** 90-101 (2015).

15. Schuller. H. M. Is cancer triggered by altered signalling of nicotinic acetylcholine receptors? *Nat. Rev. Cancer* **9.** 195-205 (2009).

16. Schamberger. A. C. *et al.*. Cigarette smoke-induced disruption of bronchial epithelial tight junctions is prevented by transforming growth factor-b. *Am. J. Respir. Cell Mol. Biol.* **50.** 1040-1052 (2014).

17. Lemjabbar. H. *et al.* Tobacco smoke-induced lung cell proliferation mediated by tumor necrosis factor alpha-converting enzyme and amphiregulin. *J. Biol. Chem.* **278.** 26202-26207 (2003).
